# Microenvironment shapes *Staphylococcus aureus* phenotypes in children with cystic fibrosis over time

**DOI:** 10.1016/j.isci.2026.117708

**Published:** 2026-10-01

**Authors:** Stefania A. Robaldi, Rosana Pereda, Maria Constanza Pautasso, Enrique Blasko, Ailen Fretes, Trinidad Jaureguialzo, Mateo Diaz Appella, Mariana B. Galeano, Maria Jose Rial, Silvina Smith, Eugenia Ginestet, Norma Gonzalez, Javier M. González, Andrea Smania, Alejandro J. Vila, Claudia Sola, Pablo M. Cassanelli, Paula M. Tribelli

**Affiliations:** 1 Universidad de Buenos Aires, Facultad de Ciencias Exactas y Naturales, Buenos Aires, Argentina; 2 IQUIBICEN-CONICET, Buenos Aires, Argentina; 3 Hospital General de Niños “Pedro de Elizalde”, Buenos Aires, Argentina; 4 CIBICI-CONICET, Córdoba, Argentina; 5 INBIONATEC, CONICET-UNSE, Santiago del Estero, Argentina; 6 Universidad Nacional de Córdoba, Facultad de Ciencias Químicas, Departamento de Química Biológica Ranwel Caputto, Córdoba, Argentina; 7 CONICET, Universidad Nacional de Córdoba, Centro de Investigaciones en Química Biológica de Córdoba (CIQUIBIC), Córdoba, Argentina; 8 Instituto de Biología Molecular y Celular de Rosario (IBR, CONICET-UNR), Rosario, Argentina; 9 Facultad de Ciencias Bioquímicas y Farmacéuticas, Universidad Nacional de Rosario, Rosario, Santa Fe, Argentina

**Keywords:** *Pseudomonas aeruginosa*, *Staphylococcus aureus*, cystic fibrosis, diversity, persistent infection

## Abstract

*Staphylococcus aureus* is a common pathogen in children with cystic fibrosis (CF), frequently preceding or coexisting with *Pseudomonas aeruginosa*. The impact of transient *P. aeruginosa* infection and therapeutic intervention on *S. aureus* phenotypic shaping remains poorly understood. We characterized 546 isolates from a longitudinal cohort of sixteen pediatric patients in CF follow-up, either monoinfected or coinfected, over eighteen months. Isolates were grouped into three phenotypic clusters. Monoinfected patients were enriched in a cluster with high pigment and biofilm, whereas transient *P. aeruginosa* detection was associated with a shift toward reduced pigment and biofilm, while coinfected patients maintained stable phenotypes. Phenotypes were determined by the airway microenvironment rather than by clonal background. In two monoinfected patients, this shift coincided with transient infection and ciprofloxacin treatment with increased MRSA; *in vitro*, MRSA predominated over MSSA when both *P. aeruginosa* and ciprofloxacin were present, underscoring the need to monitor *S. aureus* phenotypic transitions.

## Highlights

• 546 *S. aureus* isolates from CF patients grouped into three phenotypic clusters
• Phenotypes were determined by airway microenvironment, not clonal genetic background
• Transient *P. aeruginosa* shifts *S. aureus* toward reduced pigment and biofilm
• Ciprofloxacin and transient *P. aeruginosa* favored MRSA over MSSA in cocultures

## Graphical abstract

**Figure:**
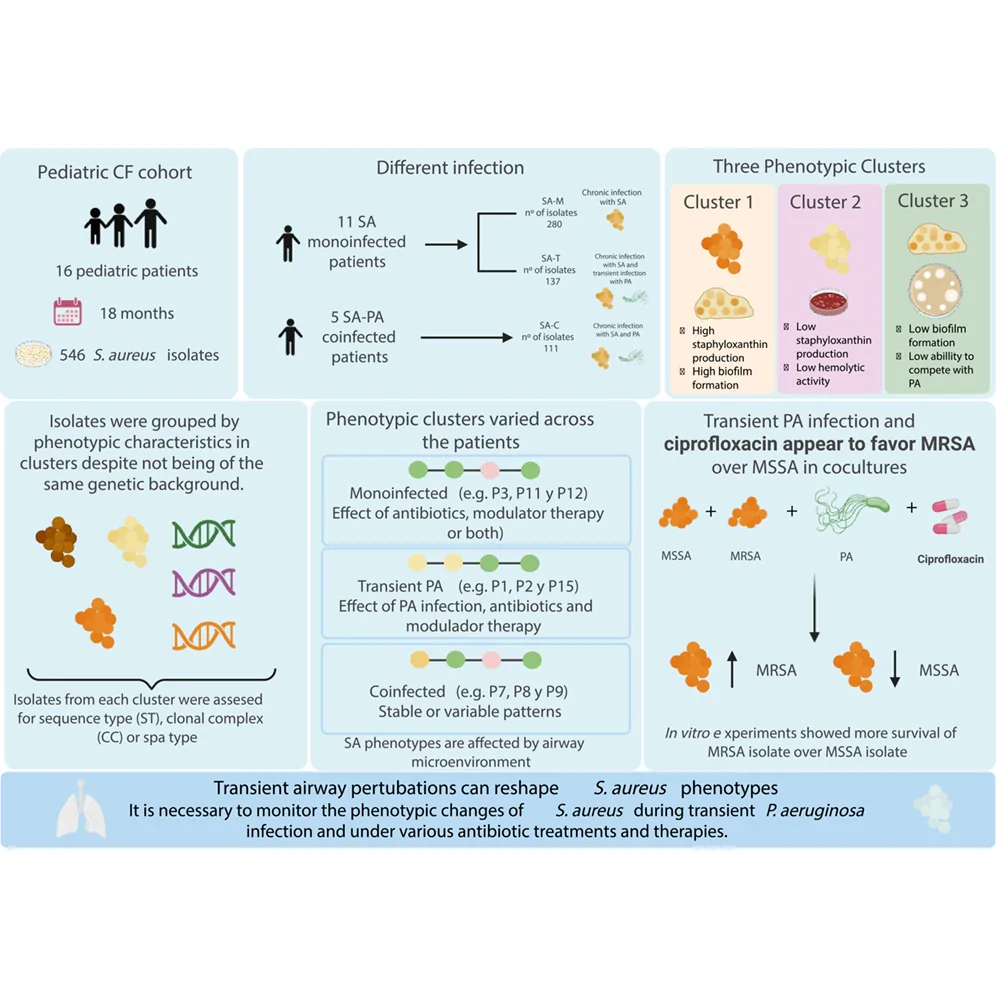

## Teaser

Disease; Fibrosis

## Introduction

Cystic fibrosis (CF) stands as a frequent genetic disorder in the Caucasian population, caused by mutations in the CF transmembrane conductance regulator (CFTR) gene. The disease affects numerous organs and leads to various complications associated with increased morbidity and mortality. Examples include cystic fibrosis liver disease (CFLD) and CF-related diabetes.^1^ Chronic airway infections represent a leading cause of morbidity and mortality in these patients, with the burden being particularly severe in low- and middle-income countries where access to specialized care and appropriate antimicrobial therapy is limited.^2^ Although CFTR modulators reshaped the CF clinical scenario, bacterial infections, and consequently its surveillance and treatment, remain critical due to treatment limitations such as age restrictions, CFTR mutation, patient nutritional condition, and drug cost and accessibility.^3^

In individuals with CF, the pulmonary microbiome typically manifests dysbiosis alongside a pro-inflammatory milieu, leading to recurrent infections that may either be a consequence or a contributing factor, ultimately progressing to a chronic state over time.^4^ *Staphylococcus aureus* is commonly acknowledged to be the main infective agent during childhood, while *Pseudomonas aeruginosa* tends to prevail in patients as they age.^1^^,^^5^ Infections during childhood are important for CF patients since they provoke a progressive damage to lung tissue, decreasing the respiratory function and therefore having an early deleterious impact in their lives.

*S. aureus* and *P. aeruginosa* possess a plastic metabolism and a set of virulence factors including quorum sensing molecules, biofilm formation, extracellular enzymes, and particularly *S. aureus* possess several toxins including hemolysins and immune system evasion proteins.^6^ The interaction between these bacterial species is complex, ranging from competition, involving *P. aeruginosa* attractants and antistaphylococci factors secreted by *S. aureus* and *P. aeruginosa*, respectively) to coexistence and even metabolic cooperation.^7^^,^^8^^,^^9^ Although these two bacterial species have been isolated from patients, and their coexistence has also been reported *in vitro*,^10^^,^^11^ it remains unclear how this coexistence influences the selection of certain *S. aureus* phenotypes *in vivo*, particularly over time. We wanted to know how the presence of *P. aeruginosa* and airway microenvironment—altering interventions select for specific *S. aureus* phenotypes.

To address this question, we assembled a collection of 546 *S. aureus* isolates obtained through a rational, prospective longitudinal study of 16 pediatric patients with a CF diagnosis. The isolates were classified as *S. aureus* monoinfected or *S. aureus-P. aeruginosa* coinfected, enabling us to investigate the phenotype pattern of *S. aureus* isolates exposed to different airway microenvironments (including modulator and antibiotic therapies). This study revealed that the phenotype of *S. aureus* is mostly determined by the airway microenvironment rather than by its clonal background, with an unexpected observation showing enhanced *in vitro* survival of resistant phenotypes.

## Results

### Phenotypical characterization of *S. aureus* isolates from pediatric cystic fibrosis patients

All patients were chronically infected with *S. aureus* (SA) and were classified as SA-monoinfected (*N* = 11) and as SA-PA-coinfected patients (*N* = 5). Clinical and microbiological characteristics of the 16 pediatric patients included in the study are summarized in Table S1. The median number of SA isolates obtained per patient was 30.5 isolates (IQR: 20.5–44.5, Table S1). No small-colony variant phenotype was isolated during the sampling period.

We analyzed a total of 546 SA isolates collected between July 2021 and January 2023. Given the dynamic nature of infections during the sampling period, SA isolates were classified as follows: SA-M, isolates from patients recruited as monoinfected who did not develop PA infection or were collected before a transient PA isolation; SA-T, isolates obtained during or after a transient PA infection in monoinfected patients; and SA-C, isolates from patients recruited as coinfected (Figure 1) in which the co-infecting PA isolates showed a mucoid phenotype typically observed in chronic infections.

**Figure 1:**
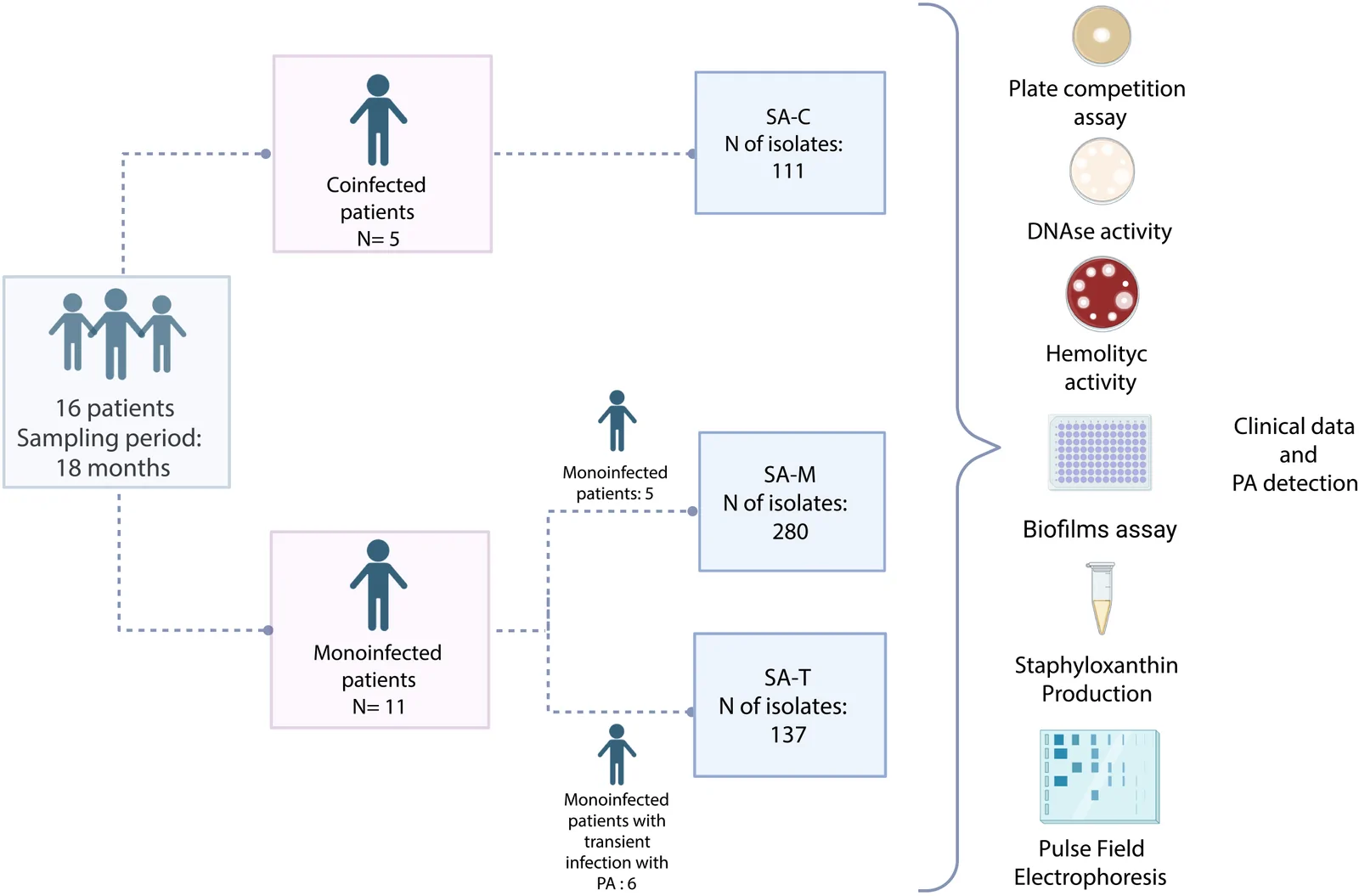
Clinical sampling design Sixteen patients with CF diagnosis were classified as mono-infected or co-infected according to chronic infection status with SA or SA-PA. Isolates of SA and PA were collected from respiratory samples from these patients.

As a first approach, we analyzed the different phenotypes (biofilm formation, competition with the reference PAO1 strain, hemolytic and DNAse activity, and staphyloxanthin production) individually, using the SA classification mentioned previously. The median of competition ability and biofilm did not show any differences among groups (Figures 2A and 2B). Hemolysis agar plate assay showed differences between SA-M/SA-C and SA-T/SA-C (Figure 2C). Quantitative hemolytic assay using sheep erythrocytes was performed for a randomly selected isolates which showed results than the agar plate assays (Table S2) supporting the result for the entire set of isolates. To further analyze this phenomenon, we performed a Chi Square-test was conducted using the agar plate assays using the 546 isolates. We showed differences in hemolytic activity between the SA-M and SA-C group with higher proportion of non-hemolytic isolates in SA-M (***χ***^2^ = 3.860, df = 1, *p* value = 0.0494, 16.78% in SA-M vs. 9.01% in SA-C) (Figures 2C and 2D). Staphyloxanthin content differed moderately among groups being the lowest in the SA-C (SA-C median = 0.1450 *n* = 111, SA-M median = 0.1830 *n* = 280 and SA-T median = 0.2580 *n* = 137) (Figure 2E). Extracellular DNAse activity showed higher values for the SA-M group and the lowest for SA-C group, although the effect size was small (Figure 2F).

**Figure 2:**
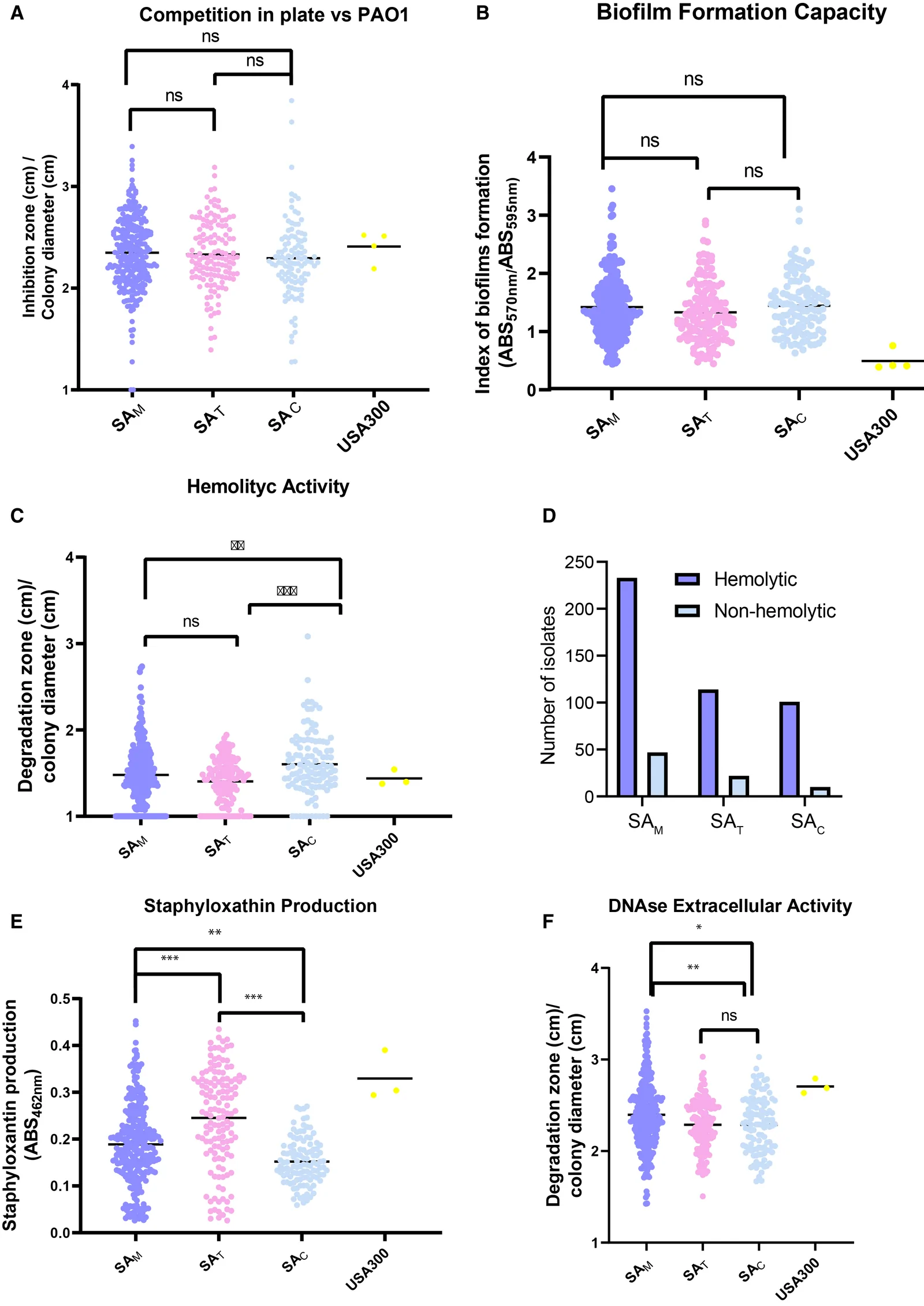
Phenotypes of SA isolates (A) Competition assay against *P. aeruginosa* PAO1 reference strain. (B) DNAse activity in DNA-agar plates. (C) Hemolytic activity in sheep agar blood. (D) Non-hemolytic isolates proportion. (E) Staphyloxanthin production obtained after methanol extraction. (F) Biofilm formation in polystyrene microplates determined by crystal violet stain. SA USA300 was included as additional control. Each dot represents the mean value obtained for an individual clinical *S. aureus* isolate. Horizontal lines indicate the mean ± (SEM/SD) for each group. Statistical comparisons among groups were performed using one-way ANOVA followed by Tukey’s test. *p* < 0.05, ∗*p* < 0.01, ∗∗*p* < 0.001, and ∗∗∗*p* < 0.0001.

To better characterize the correlation between some of the phenotypes, we performed hierarchical clustering based on a principal component analysis (PCA) using the isolates’ phenotypic data generated in the *in vitro* assays described previously (Figure S1; Tables S3 and S4). As is shown in Figures 3A–3C, SA isolates could be classified in three defined clusters named from cluster 1 to 3. Each cluster showed a similar number of assigned isolates (cluster 1 = 199, cluster 2 = 139, and cluster 3 = 188) and all of them presented contributions of different patients (Figures 3B and 3E). Cluster 1 was characterized by high staphyloxanthin and biofilm production. Additionally, the competition ability was higher in comparison with other groups (Figures 3B and 3C). The most relevant characteristic of cluster 2 was the low hemolytic activity and low staphyloxanthin production (Figures 3B and 3C). Cluster 3 isolates showed low biofilm formation and low ability to compete with PA as well as high DNAse production (Figure 3C). We sought to identify the factors that determine the phenotypes underlying the observed clustering. To this end, we analyzed the association of each cluster (in terms of the number of isolates) with the SA group (M, T, or C), and other factors. Although not significant, the association between clusters and SA groups (SA-M, SA-T, or SA-C) showed a trend in which cluster 1 was associated mainly with SA-C, while cluster 3 was associated with SA-M and SA-T (Figure 3F). Cluster 2 showed no evident association with these groups. Our results also showed that cluster 3 had an enrichment of SA isolates from patients using the modulator therapy at any point during the sampling period (Fisher test, *p* < 0.01; Figure 3G), whereas the presence of chronic PA did not show any association with a specific cluster (Figures 3B and 3H). However, within cluster 1, a subgroup characterized by high staphyloxanthin production showed no cases of chronic PA (Figure 3B). Furthermore, co-isolation with *Candida* spp. was found to be rarer in cluster 3 (Fisher test, *p* < 0.05) whereas SA co-isolated with *Aspergillus* spp. more frequently in cluster 2 (Fisher test, *p* < 0.0001), highlighting the potential role of fungal species in terms of microenvironment-associated SA phenotypes.

**Figure 3:**
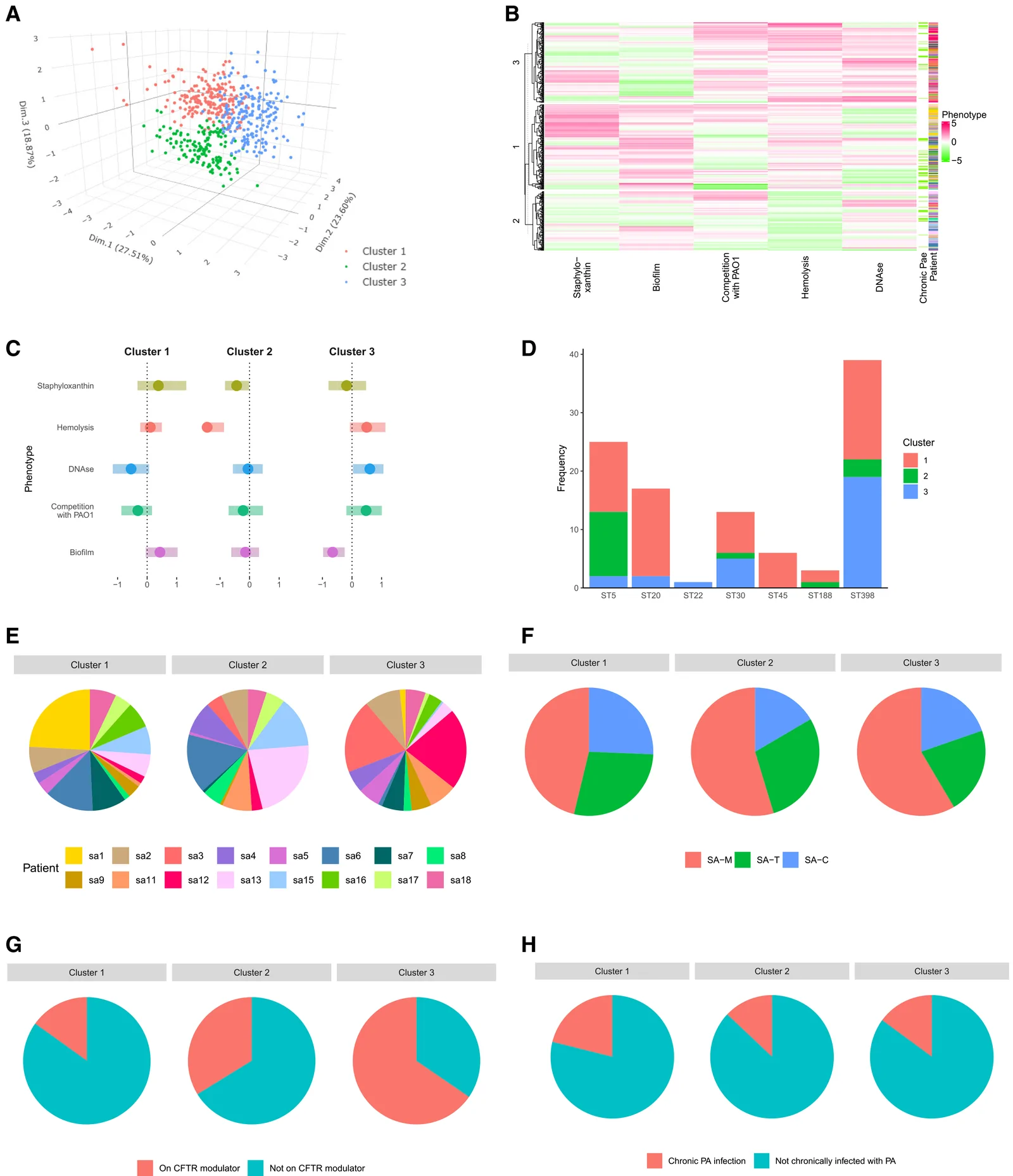
Phenotypic clustering of SA isolates (A) Principal components analysis (PCA) based on the SA clustering. (B) Hierarchical clustering analysis. Different phenotypes, presence of chronic PA and patient origin are shown. Colors indicate the median of the individual isolates. (C) Representation of phenotypic profiles for each cluster. The zero represents the median of each phenotype calculated with the 546 analyzed isolates. (D) Frequency of each ST for the three observed clusters for the analyzed isolates using pulse field electrophoresis analysis. (E) Proportion of isolates belonging to different clusters in each recruited patient. (F) Proportion of isolates belonging to different clusters in the SA defined categories (SA-M, SA-T, and SA-C). (G) Proportion of isolates belonging to different clusters in patients using the modulator CF therapy or not. (H) Proportion of isolates belonging to different clusters in patients chronically infected with PA.

Additionally, we analyzed whether the clusters were composed of specific SA lineages or clonal complexes (CCs). To investigate this, we selected representative isolates from different clusters belonging to the same patient and performed pulsed-field gel electrophoresis (PFGE), complementing with molecular *spa* or multilocus sequence types (MLST) typing for representative isolates of the obtained pulse-types (appendix Figures 3A and 3B; appendix Table S5). Our results primarily showed a diversity of SA lineages within each cluster, including ST398 (CC398), ST5, ST20, ST30 (CC30), ST22 (CC22), ST45 (CC45), and ST188 (CC1), highlighting the genetic variability among patients and even within individual patients. MSSA CC398 appeared to be the most frequent lineage among the recruited patients (Table S5). PFGE profiles within individual patients were variable; for example, patient 1 (hereinafter P1) consistently showed ST20 isolates with the same *spa* type across samples, until transient PA infection, where we found PFGE and *spa* variations corresponding with the appearance of ST30 strains. In contrast, other patients such as P9 and P18 exhibited different STs—ST45 and ST398 in P9, and ST5 and ST398 in P18. Isolates with identical *spa* and sequence type (ST) showed different phenotypic profiles across patients. For instance, in P12, P9, and P18, isolates with *spa* 1451/ST398 fell into different clusters depending on the patient. The same occurred for P1, P2, and P9, where isolates with the same molecular type were assigned to distinct phenotypic clusters (Table S5).

Our results showed that phenotypic clustering was not determined by the ST, as different STs were distributed across distinct clusters (Figure 3D). Similar findings were observed when individual phenotypes were analyzed, indicating that specific phenotypic values were not associated with a particular lineage.

### SA phenotypes are affected by airway microenvironment

Infection in CF pediatric patients is highly dynamic. Different factors can affect the airway environment, not only by the presence of different microorganisms but also due to therapeutic intervention. For example, patients recruited as coinfected may have initiated triple therapy with elexacaftor/tezacaftor/ivacaftor (ETI) during the sampling period, or an SA-monoinfected patient may have experienced a transitory PA infection (Table S1). To address how these factors influence the selection of SA phenotypes, we studied SA isolates from representative patients over time, focusing on PA decrease or transient infection, initiation of modulator therapy and antibiotic treatment. First, we analyzed monoinfected patients P3, P11, and P12 which showed all SA-M (Figure 1). In P3, a stable cluster 3 pattern was observed, except for a single time point in May 2022, which coincided with empiric ciprofloxacin treatment due to a worsening of clinical parameters, although no Gram-negative bacteria were isolated (Figure 4A). P11, is a child who underwent changes in modulator therapy. At the time of recruitment, the patient was receiving lumacaftor/ivacaftor, which was replaced by ETI in January 2022. Five months later, SA isolates displayed variable phenotypes, with a shift from cluster 2 to cluster 3, which was maintained in subsequent samplings (Figure 4B). In P12, isolates mainly belonged to cluster 3. This patient underwent antibiotic treatment around August 2022 due to lung function deterioration—initially with oral ciprofloxacin and trimethoprim-sulfamethoxazole (TMS), followed by two hospitalizations requiring intravenous amikacin and vancomycin. The isolates obtained during and after these antibiotic treatments exhibited phenotypic changes, including decreased hemolysis and biofilm formation (Figure 4C).

**Figure 4:**
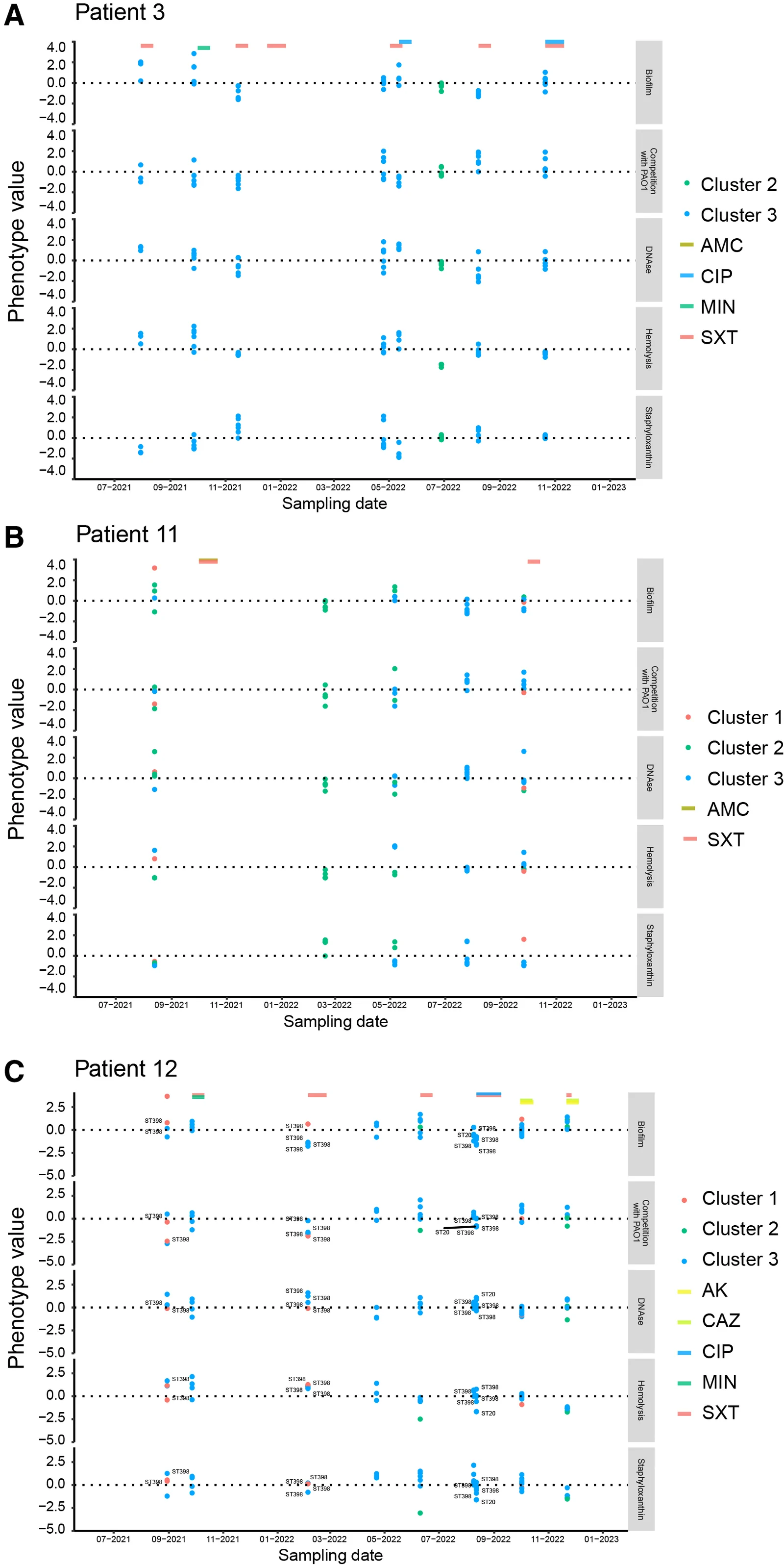
Temporal dynamics of the phenotype values in SA isolates from P3, P11, and P12 All isolates are part of SA-M group. Each data point represents the normalized mean value from three independent determinations of biofilm formation, competition with PAO1, DNAse activity, hemolysis, or staphyloxanthin production for a single isolate. At each sampling time point, multiple isolates were randomly selected from patients undergoing routine clinical monitoring or experiencing exacerbations. AMC, amoxicillin/clavulanic acid; AK, amikacin; CAZ, ceftazidime; CIP, ciprofloxacin; MIN, minocycline; SXT, trimethoprim/sulfamethoxazole.

We also analyzed three monoinfected patients who experienced transient PA infections. From these individuals, we obtained SA-M isolates (collected before the PA infection) and SA-T isolates (collected during and after the transient PA infection) (Figure 1). In P1, most SA-M isolates belonged to cluster 1; however, when a transient PA infection occurred, together with oral ciprofloxacin and inhaled colistin treatment, SA-T isolates shifted to cluster 3, exhibiting lower biofilm formation and staphyloxanthin production, while the other phenotypes remained largely unchanged (Figure 5A). P2 experienced a transient PA infection accompanied by ciprofloxacin treatment combined with inhaled tobramycin, which was associated with an increase in SA-T isolates belonging to cluster 2 (Figure 5B). In September 2022, this patient began inhaling DNAse therapy, and from that point onward, all isolates grouped in cluster 3 (Figure 5B). P15 was recruited as monoinfected but subsequently experienced a nearly year-long PA infection. During the initial period, SA-M isolates belonged to cluster 1, and after approximately four months, the SA-T isolates were classified into cluster 2 (Figure 5C). Additionally, this patient initiated ETI in June 2022. While PA was no longer isolated, SA continued to be recovered, displaying a progressive decrease in hemolysis even after PA clearance, whereas biofilm formation and staphyloxanthin levels were restored to earlier values (Figure 5C).

**Figure 5:**
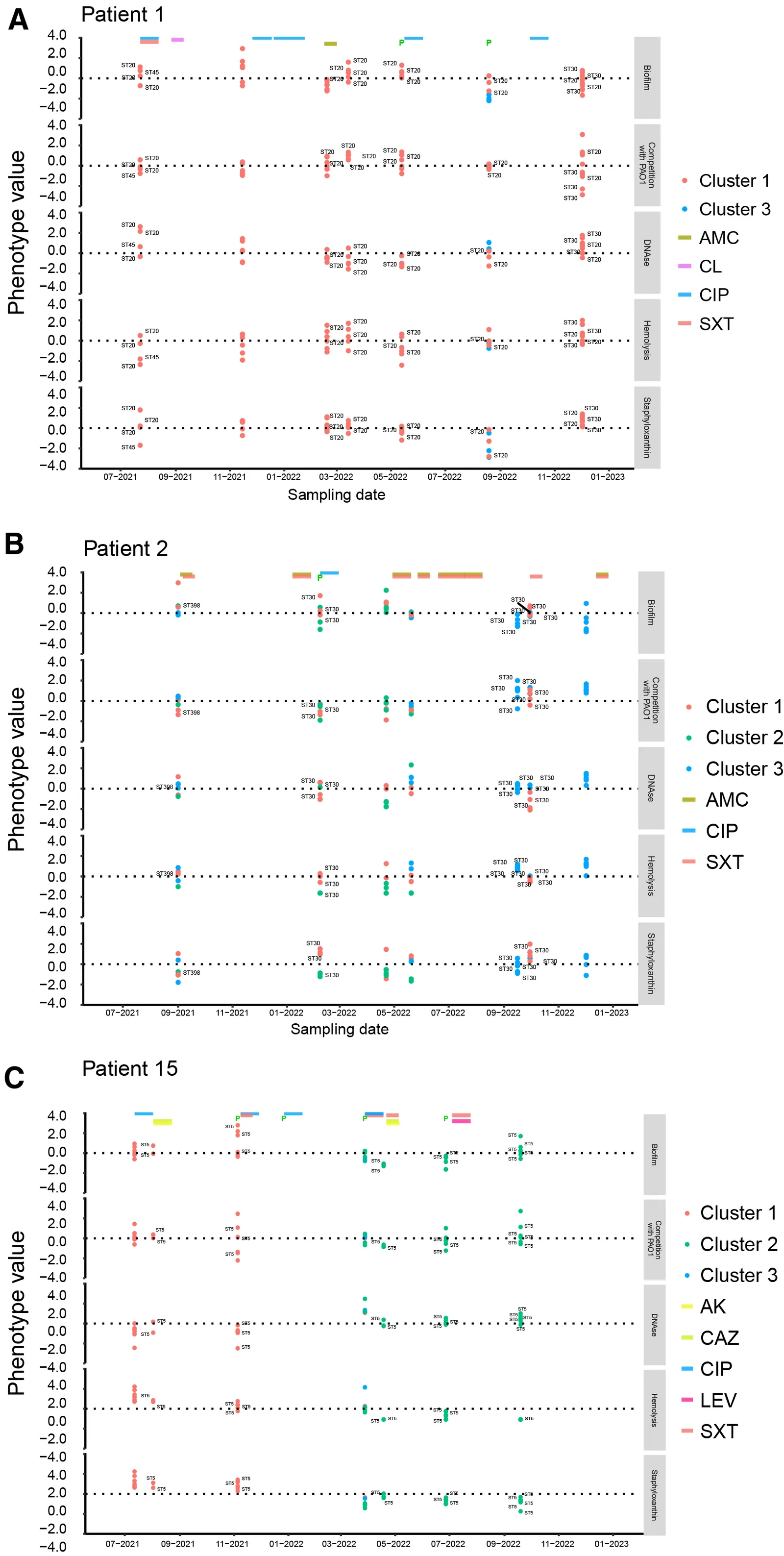
Temporal dynamics of the phenotype values in SA isolates from P1, P2, and P15 All isolates are part of SA-M and SA-T groups. Each data point represents the normalized mean value from three independent determinations of biofilm formation, competition with PAO1, DNAse activity, hemolysis or staphyloxanthin production for a single isolate. At each sampling time point, multiple isolates were randomly selected from patients undergoing routine clinical monitoring or experiencing exacerbations. AMC, amoxicillin/clavulanic acid; AK, amikacin; CAZ, ceftazidime; CIP, ciprofloxacin; CL, cephalexin; LEV, levofloxacin; SXT, trimethoprim/sulfamethoxazole.

Finally, for coinfected patients, we selected P7, P8, and P9 for longitudinal analysis of SA-C isolates. P7 began ETI after April 2022 and prior to modulator therapy, SA-C isolates were grouped into clusters 1 and 3, with cluster 1 being the most frequent. After April 2022, PA was no longer isolated, while SA isolates were predominantly assigned to cluster 3, displaying altered hemolytic activity and more variable biofilm formation (Figure 6A). For P8 and P9, we observed a different phenomenon, probably due to SA displacement or underdiagnosis caused by the presence of PA. Sampling from P8 was characterized by the absence of SA for several months. When SA isolates reappeared, a mixture of isolates belonging to the three clusters was observed. However, less than two months later, all SA isolated were classified as cluster 2 by our algorithm (Figures 6B and 6C).

**Figure 6:**
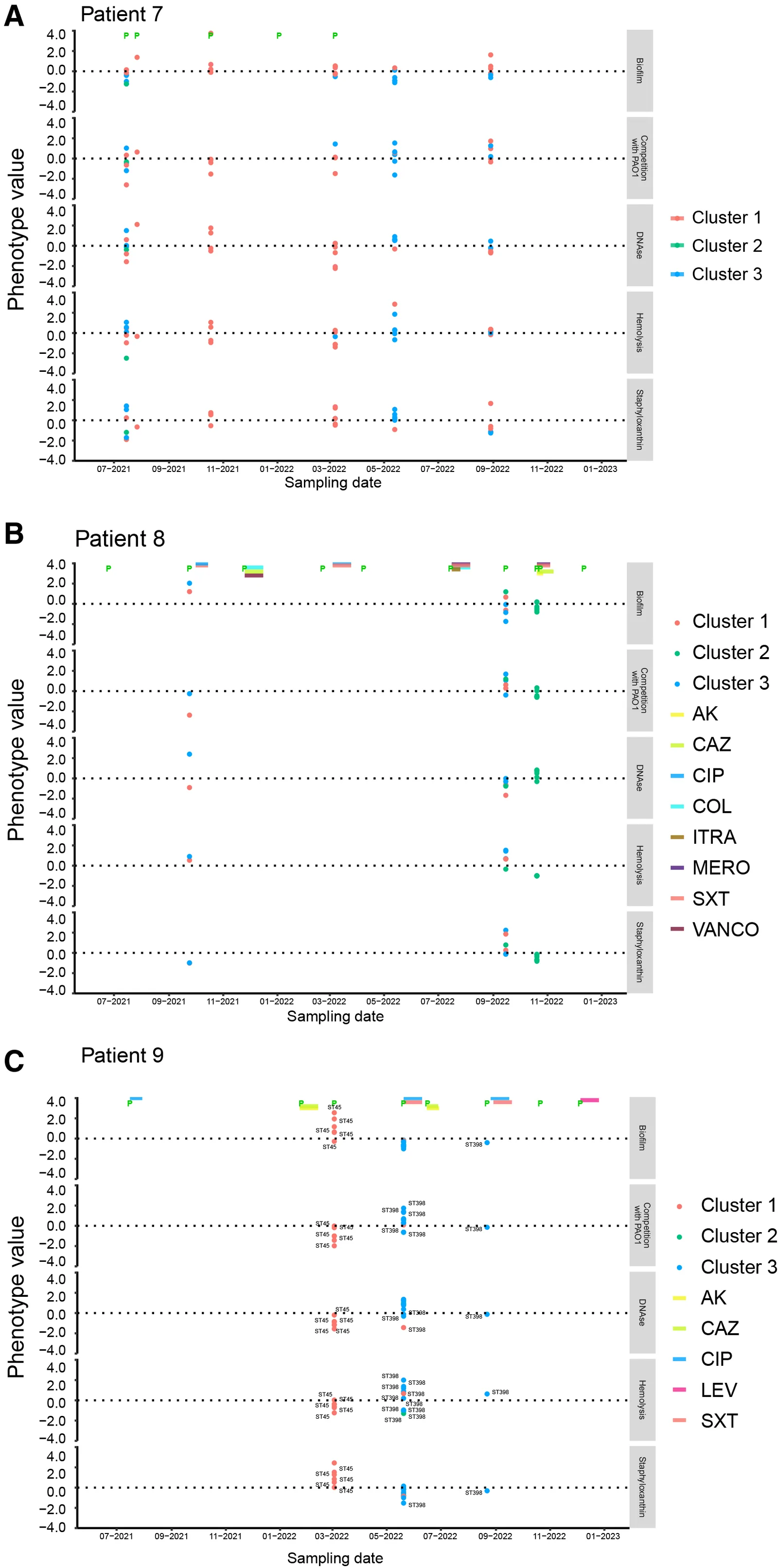
Temporal dynamics of the phenotype values in SA isolates from P7, P8, and P9 All isolates are part of SA-C group. Each data point represents the normalized mean value from three independent determinations of biofilm formation, competition with PAO1, DNAse activity, hemolysis, or staphyloxanthin production for a single isolate. At each sampling time point, multiple isolates were randomly selected from patients undergoing routine clinical monitoring or experiencing exacerbations. AK, amikacin; CAZ, ceftazidime; CIP, ciprofloxacin; COL, colistin; ITRA, itraconazole; MERO, meropenem; LEV, levofloxacin; SXT, trimethoprim/sulfamethoxazole; VANCO, vancomycin.

Overall, our results show that the SA phenotype clustering over time varied in response to fluctuations in PA presence, initiation of modulator therapy, or antibiotic treatment targeting Gram-negative bacteria such as ciprofloxacin. These changes were not associated with the SA clonal complex but rather with this varying microenvironment (Figures 4, 5, 6, and S2–S8).

### Transient *P. aeruginosa* infection and ciprofloxacin treatment favor MRSA over MSSA

MRSA represents a clinical threat in children with CF and is routinely reported by the hospital’s clinical microbiology laboratory using standard clinical methods. In some patients who experienced a transient *P. aeruginosa* infection treated with ciprofloxacin a shift from MSSA to MRSA was reported by the hospital’s laboratory.

Accordingly, we tested the proportion of methicillin-resistant isolates in SA-M and SA-T from the following patients: P1, P2, P4, P5, and P15 (Table 1), using resistance to FOX as a surrogate marker according to CLSI guidelines.

**Table 1:** MRSA and MSSA proportion

Patient	Number of isolates	% Of isolates			
		SA-M MRSA	SA-T MRSA	SA-M MSSA	SA-T MSSA
Patient 1	48	0	14.6	37.5	47.9
Patient 2	45	0	77.8	13.3	8.9
Patient 4	29	0	0	0	100
Patient 5	18	16.7	5.6	5.6	72.3
Patient 15	35	0	0	22.9	77.3

MRSA and MSSA proportion in SA-M and SA-T from patients with PA transitory infection and treated with ciprofloxacin.

In P4 and P15, no isolates presented methicillin resistance, while in P5 the proportion of MRSA was low and similar for SA-M and SA-T groups (Table 1). For P1 and P2, a Fisher’s exact test revealed a significant increase in the percentage of MRSA isolates, rising from 0% to 14.6% and 0%–77.8%, respectively, after *P. aeruginosa* infection and ciprofloxacin treatment, consistent with medical records (Table 1, Fisher test *p* < 0.001). Interestingly, P1 and P2 were under 6 years old of age, and no modulator therapy was used for their cases. On the other hand, P5 and P15 used CF modulators, and these patients, together with P4, were between 9–14 years old. Changes from MSSA population to MRSA lineages were not observed during chronic PA infection despite the different applied therapies.

Transient PA infections often originate from environmental strains which exhibit a more virulent phenotype than those involved in chronic infections, including a higher production of antistaphylococcal factors like LasA and pyocyanin.^12^ Thus, we hypothesized that PA involved in transient infections exhibit environmental-like traits that, in combination with ciprofloxacin, favor the survival of MRSA isolates over MSSA. To test this hypothesis, we employed the *P. aeruginosa* PA14 strain (PA14), a highly virulent isolate with robust pyocyanin production, resembling environmental or transient *P. aeruginosa*. To select *S. aureus* isolates for *in vitro* experiments, we first performed standard minimum inhibitory concentration (MIC) assays. One MRSA and one MSSA isolate, both from P2 and showing a 0.50 μg/mL MIC of ciprofloxacin, were selected and incubated either alone or in co-culture with PA14, in the presence or absence of a subinhibitory concentration of ciprofloxacin (0.25 μg/mL) (Table S6; Figure S9). MRSA and MSSA showed comparable CFU/mL in control monocultures and when exposed to PA14 or ciprofloxacin individually (Figure S10). However, in co-cultures containing MRSA + MSSA + PA14 with ciprofloxacin supplementation, we observed a marked increase in the proportion of MRSA, an effect not detected in the absence of the antibiotic (Figure 7). We performed the same experiment using a clinical isolate of PA from P1 (hereinafter PA_T_), which corresponded to a transitory infection, reasoning that would retain more virulence factors similar to PA14 compared to an isolate from a chronic infection. Additionally, we conducted the experiment using a chronic isolate from P9, a coinfected patient (hereinafter PA_C_). We showed that MRSA has a higher survival than MSSA in presence of ¨both clinical PA isolates and the presence of ciprofloxacin in the PA_T_ the proportion still was higher (Figure 7). In overall, our *in vitro* results showed that in presence of PA within a heterogeneous population of SA, like we observed in our sampling, the MRSA had a higher survival than MSSA, opening questions regarding the dynamic of polymicrobial infections.

**Figure 7:**
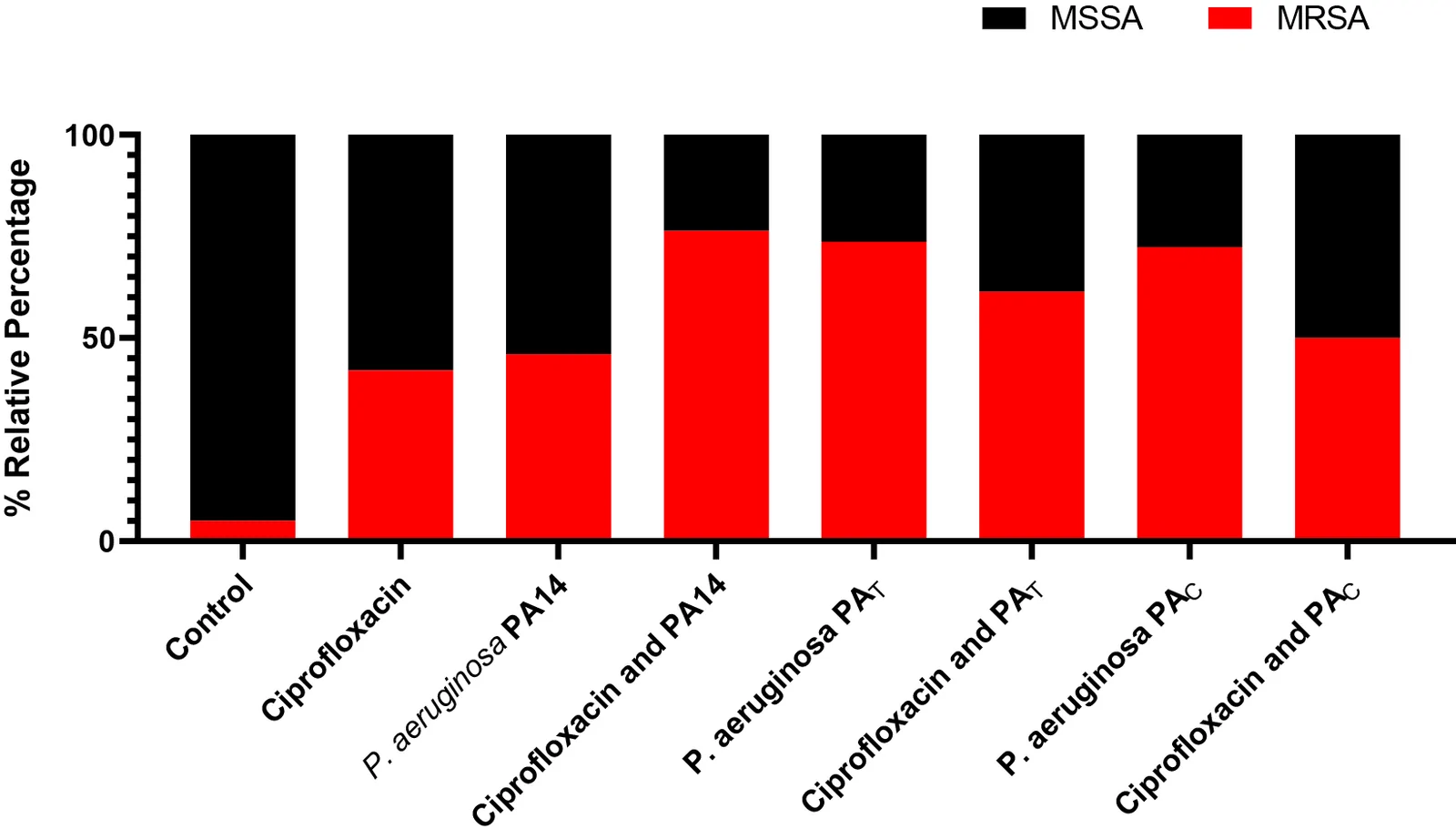
Impact of PA14, PA_T_ and PA_C_in MSSA and MRSA isolates survival in presence and absence of ciprofloxacin The percentage of MRSA or MSSA after cultivation was determined by plating followed by replica plating in oxacillin plates.

## Discussion

Polymicrobial infections can lead to a worse patient prognosis and are affected by a variety of factors, including the pathological scenario, the host immune system, the genetic background of circulating strains, the native microbiome, the antibiotic and other drug therapies and the presence of different transient or chronic, opportunistic or pathogenic microbial species.^13^^,^^14^^,^^15^^,^^16^

CF is a genetic disease characterized by an inflammatory airway environment and bronchiectasis, with respiratory deterioration requiring lung transplant and ultimately leading to death. CF has been the hallmark of chronic polymicrobial infections, with an initial colonization of SA during childhood, while subsequent PA infection can lead to a coinfection scenario or displacement of SA^17^^,^^18^ However, differences in colonization dynamics and life expectancy can be observed across countries, depending on socioeconomic factors such as access to health care and early diagnosis.^19^^,^^20^ Nowadays CF modulator therapy has changed the overall clinical outlook for the patients, but there are still limitations related to CFTR mutations, patient age, metabolic profile, and economical access to the treatment.^21^ Despite improvements in lung function and increased life expectancy, the presence of pathogenic bacterial species such as SA and PA is still a concern.^22^ In this study we recruited 16 pediatric patients from a hospital in Buenos Aires, Argentina, with different CFTR mutations and a variety of clinical profiles, including mild, moderate, and severe lung function impairment. Additionally, the cohort included patients of different ages and microbiological profiles: young children with recently acquired chronic SA infection, and older children or adolescents with either chronic SA infection or SA-PA coinfection, with episodic isolation of other microorganisms.

Bacterial interactions are complex: PA and SA display different behaviors ranging from antagonism to metabolic syntropy in CF patients mediated by the acetoin metabolism, as well as cooperation in wounds through a division of labor.^6^^,^^22^ More complicated scenarios arise when the initial colonizer is considered. The presence of SA as the initial colonizer in CF, may increase the cost of infection for PA, which in turn increases the expression of virulence factors to cope with the competitor.^23^

Rumpf et al.^24^ performed an exhaustive analysis of different longitudinal studies in CF patients, examining mutation rates, gene transference, gene expression, gene regulation, and phenotypic heterogeneity. These authors highlighted the importance of analyzing more than one gene to understand the SA virulence. Here we followed the SA infection dynamics of pediatric CF patients using five different phenotypes (biofilm formation, staphyloxanthin production, competition ability against PAO1, DNase activity, and hemolytic activity), which allowed us to group the SA isolates into clusters displaying a different phenotypic pattern and to identify trends that helped predict infection dynamics.

Our results showed that the SA isolates were grouped in three clusters in which changes in hemolysis, biofilm, and staphyloxanthin production seemed to be relevant factors in determining regrouping after disturbing events such as transient PA infection or CF modulator therapy initiation. We observed a shift from cluster 1 toward cluster 2 and cluster 3 that possesses a pattern with lower biofilm formation and staphyloxanthin production after transient PA infection. Staphyloxanthin is a triterpenoid embedded in the cell membrane, and its biosynthesis depends on the presence of the crtOPQMN operon, whose expression is driven by a single promoter. Contradictory results can be found in literature regarding pigment production and its protective role against pyocyanin-derived oxidative stress.^25^^,^^26^ We found that staphyloxanthin production was higher in SA-M and SA-T compared with SA-C. Besides pigment production, we analyzed biofilm formation in *SA*, which depends on exopolysaccharide production derived from the *ica* operon, but also on the contribution of proteins and adhesins (Fbn, clumping factors and Spa). Interestingly, SA biofilm formation, as well as staphyloxanthin production, is positively regulated by the regulators SarA and SigB. A recent study showed that coculture of clinical *S. aureus* and *P. aeruginosa* isolates produces transcriptomic responses that differ substantially from those induced by the reference strain PAO1, particularly in biofilm-associated pathways.^27^ Consistent with this, *P. aeruginosa*-derived factors can directly repress *spa* expression in SA.^5^ Hemolytic activity is crucial during SA infection leading to red blood cells lysis and hemoglobin releasing as iron source. Isolates from cluster 2 showed a higher proportion of non-hemolytic strains. This cluster comprises different types of SA isolates (SA-M, T, or C) without a clear association with PA. The expression of genes encoding hemolytic toxins are positively regulated by Agr and negative *agr* variants have been reported in CF isolates before.^24^

Regarding the SA background, pulsed-field electrophoresis and complemented molecular typing performed in this study showed a variety of SA CCs involved in CF infections, including CC1, CC5, CC45, CC22, CC30, and CC398 and no small-colony variant was detected through our sampling. These lineages have been reported as causative agents of bloodstream infections in Argentina and other South American countries.^28^ In Argentina, CC398 represents the most frequent MSSA lineage and has been linked to a 23.4% increase in SA infections, particularly community onset infections, over the past decade,^29^ while CC5 and CC30 constitute the predominant MRSA CCs.^28^^,^^29^ This contrasts with epidemiological data from the United States, where more than 80% of SA isolates belong to CC5, CC8, CC30, and CC45, while CC398 appears to be irrelevant among MSSA in both non-CF and CF patients,^30^^,^^31^ suggesting that CF infections by SA parallel the lineages circulating in the community. However, the scenario appears to be more complex, as another survey reported an association of CF isolates with hospital-acquired SCCmec, probably indicating some role of the hospital environment.^32^ In a previous study, a dynamic transmission process of MRSA clones between hospitals and the community was described,^33^ supporting the notion that circulating lineages in both settings can also colonize patients with CF.

Interestingly, in our study, similar genetic backgrounds were not associated with a given cluster, highlighting the role of the microenvironment in selective pressure. Indeed, Bernardy et al.^34^ and Wieneke et al.^35^ showed phenotypic diversity within the same SA lineage in strains isolated from CF patients. In adult patients with chronic coinfection substantial phenotypic diversity in SA and its association with persistent PA and airway inflammation have been reported.^35^ Our longitudinal pediatric cohort reveals that phenotypic shifts arise in a far more dynamic context, where transient PA episodes, antibiotic exposure, and CFTR modulators act as selective pressures, reinforcing that short-term microenvironmental perturbations can shape *S. aureus* phenotypes.

Finally, we evaluated how PA and ciprofloxacin affect the survival of clinical MRSA and MSSA strains. Our results showed a higher survival of the MRSA isolate over the MSSA only when the ciprofloxacin was added at a subinhibitory concentration to co-cultures with PA14. Moreover, when we performed the same experiment with a PA_T_ from a monoinfected patient that suffered a transitory PA infection or a PA_C_ from a coinfected patient, we showed that the presence of PA_T_ and in modest level the PA_C_ the MRSA proportion was higher than MSSA in cocultures. In polymicrobial infections, PA14 exerts an antagonistic effect on SA, driving transcriptomic remodeling of the latter. Co-culture studies have shown that PA14 induces oxidative and nitrosative stress responses in *S. aureus*, while simultaneously repressing pathways linked to energy metabolism and virulence.^36^^,^^37^ Importantly, multidrug efflux pumps such as Nor family transporters are upregulated during these interactions, potentially enhancing SA tolerance to fluoroquinolones like ciprofloxacin.^38^^,^^39^^,^^40^ This is consistent with our observation that MRSA isolates displayed increased survival in PA14 + ciprofloxacin co-cultures, a phenotype that might be linked to NorC-mediated efflux. Together, these findings underscore how interspecies interactions can potentiate antimicrobial resilience beyond canonical resistance determinants.

All these aspects illustrate the complexity of the analysis. However, this longitudinal prospective study provided an integrated analysis of the dynamics of *S. aureus* infections, highlighting several key points: (1) Despite variability, SA isolates could be clustered into only 3 groups with distinct phenotypic patterns that did not depend on the ST (2) Cross-cluster shifts over time appear to be driven by changes in the airway microenvironment being PA transient infection a relevant factor (3) Transient PA infection along with ciprofloxacin appear to favor MRSA survival in mixed cultures with MSSA raising concerns about the potential selection of resistant clones previously present at low abundance or the acquisition of new resistant strains.

Overall, this study underlines the need to monitor *Staphylococcus aureus* phenotypic transitions associated with the presence of *Pseudomonas aeruginosa* or in response to therapeutic interventions more systematically to better understand their clinical relevance.

### Limitations of this study

First all participants were recruited in the same geographic zone (Ciudad Autónoma de Buenos Aires, Argentina). Second, our study is a single-center study with a modest *N* = 16. Third, this is an observational prospective study. Fourth, in older patients (never older than 16 years old at the beginning of the recruitment) where there was a long time of chronic infection before the sampling started, other factors could influence the microenvironment, including treatments, other infections and other unknown factors. The potential influence or association of sex, gender and/or ethnicity on this study could not be assessed, as these variables were not associated to the individual clinical *S. aureus* isolates. These limitations require more analysis in the future to generalize our observations; however, the *in vitro* experiments open new questions about the involved molecular mechanisms.

## Resource availability

### Lead contact

Requests for further information and resources should be directed to and will be fulfilled by the lead contact, Paula M. Tribelli, paulatrib@qb.fcen.uba.ar.

### Materials availability

This study did not generate new unique reagents.

### Data and code availability

• All data needed to evaluate the conclusions in the paper are present in the paper, or in the supplemental information.
• This paper does not report original code.
• Any additional information required to reanalyze the data reported in this paper is available from the lead contact upon request.

## Acknowledgments

We thank Dr. Sergio Nemirovsky for his invaluable advice on the statistical treatment of the data and Dr. Nancy Lopez for reviewing the manuscript. This work was funded by “Redes Tecnológicas de Alto Impacto, Red REPARA” program (Jefatura de Gabinete de Ministros, Argentina) to A.J.V., A.S., J.M.G, and P.M.T., the National Agency for Scientific and Technological Promotion (ANCPyT) PICT-2021-GRFTI-00265 and +4I program FCEyN-UBA to P.M.T. and by the (CONICET-PIP-2022) and the ANPCyT–PICT 2020-02171 to C.S., S.A.R., M.B.G., and M.D.A. hold a doctoral fellowship from CONICET while E.B. and A.F. from ANPCyT.

## Author contributions

S.A.R., P.M.C., C.S., and P.M.T.: conceptualization, methodology, investigation, visualization, software, writing – review and editing; M.C.P., A.F., M.B.G., M.D.A., E.B., T.J., and J.M.G.: investigation and visualization; A.J.V. and A.S.: conceptualization, writing original and revised draft; N.G., R.P., M.J.R., and S.S.: conceptualization, patient recruitment and follow-up.

## Declaration of interests

The authors declare no competing interests.

## STAR★Methods

### Key resources table

**Table:** 

REAGENT or RESOURCE	SOURCE	IDENTIFIER
**Bacterial and virus strains**		

Clinical *Staphylococcus aureus* isolates	This study	Internal isolates ID’s. From A to Z
Clinical *Pseudomonas aeruginosa* isolates	This study	PA_T_ and PA_C_
*Staphylococcus aureus USA300*	Laboratory collection	ATCC BAA-1556
*Pseudomonas aeruginosa* PAO1	Laboratory collection	ATCC 15692
*Pseudomonas aeruginosa* PA14	CIQUIBIC-CONICET (Córdoba, Argentina)	GenBank: GCA_047118485.1/RefSeq: GCF_047118485.1

**Biological samples**		

Respiratory clinical samples from pediatric cystic fibrosis patients	Hospital General de Niños “Pedro de Elizalde” | Bacteriology Department	See in Table S2

**Chemicals, peptides, and recombinant proteins**		

Tryptic Soy Broth (TSB)	Oxoid	CM0129R
DNAse Agar	Britania	B0223106
Mueller-Hinton agar	Oxoid	CM0337B
Mueller-Hinton cation adjusted broth	Thermo-Fisher	T3462-05
Oxacillin Resistance Screening Agar Base	Oxoid	CM1008
Ciprofloxacin	Sigma – Aldrich	17850-5G-F
Oxacillin	Sigma - Aldrich	28221-5G
Cefoxitin Antimicrobial susceptibility discs 30 μg	Oxoid	CT0119B

**Oligonucleotides**		

sau1-hsdS1 AF primer (CC398 PCR, forward) 5′-AGGGTTTGAAGGCGAATGGG-3′	Stegger et al.^41^	N/A
CC398r1 primer (CC398 PCR, reverse) 5′-CAGTATAAAGAGGTGACATGACCCCT-3′	Stegger et al.^12^^,^^41^	N/A
spa-1113f primer 5′-TAAAGACGATCCTTCGGTGAGC-3′	Bacurdi et al.^29^^,^^33^	N/A
spa-1514r primer 5′-CAGCAGTAGTGCCGTTTGCTT-3′	Bacurdi et al.^29^^,^^33^	N/A

**Software and algorithms**		

Graph-pad Prism	GraphPad	RRID:SCR_002798
R	R Core Team^42^	RRID:SCR_001905
FactoMineR	Lê S et al.^43^	RRID:SCR_014602
Ridom SpaServer	Ridom GmbH	
ImageJ	Schneider CA et al.^44^	RRID:SCR_003070

### Experimental model and study participant details

#### Clinical sampling design

Sixteen patients diagnosed with cystic fibrosis (CF) were randomly selected and included if, at study initiation, at least two of their last four routine cultures were positive for *Staphylococcus aureus* (herein SA) and they did not meet the criteria for chronic *Pseudomonas aeruginosa* (herein PA) infection (hereafter referred to as mono-infected), or if they met the criteria for chronic infection with both microorganisms (co-infected). Mono-infected patients could nevertheless present transient or intermittent *P. aeruginosa* isolation during follow-up (Figure 1).

Chronic infection was defined according to the Argentine Society of Pediatrics guidelines for cystic fibrosis as more than half of the respiratory cultures obtained during the previous year being positive for a given microorganism, provided that at least four routine cultures had been performed during that period^45^ and determined by the Bacteriology Department of the Central Laboratory at HGNPE. Mono-infected patients could nevertheless present transient or intermittent P. aeruginosa isolation during follow-up.

#### Sampling

Isolates of *S. aureus* and *P. aeruginosa* were collected by the hospital’s Bacteriology Department from cultured respiratory samples obtained during routine clinical visits or in the event of pulmonary exacerbation over an 18-month period. For each respiratory sample collected, 5–10 different colonies of SA and PA were randomly selected (Figure 1).

#### Clinical data collection

Relevant clinical data generated during the sampling period were extracted from the digital medical records of the recruited patients, including age, sex, CFTR variant, pulmonary exacerbations, antibiotic treatments, CFTR modulator treatment initiation and other pulmonary therapies. Microorganisms isolated on routine and exacerbation cultures during this period, along with antibiotic susceptibility reports, were also registered. The mean and interquartile range age for all patients was 8.8 (6.5–13.2) years old. The mean age for *S. aureus* monoinfected patients was 7.7 (4.3–9.8) years old and the mean age for *S. aureus – P. aeruginosa* coinfected patients was 12.6 (8.7–14.8) years old. Male sex accounted for 62,5% of patients overall, with similar proportions in monoinfected (63.6%) and coinfected (60.0%) groups.

#### Ethics statement

This study was reviewed and approved by the Ethical Review Board of Children’s General Hospital Pedro de Elizalde (PRISBA 4466). All patients or their legal guardians provided informed consent to participate in the study.

#### Bacterial strains

*Pseudomonas aeruginosa* PA14 and *Staphylococcus aureus* USA300 were used as reference strains in experimental assays.

### Method details

#### Bacterial isolates and culture conditions

SA clinical isolates (*N* = 546) were identified by standard microbiological procedures. SA cultures were grown in 5 mL tryptic soy broth (Oxoid) (herein TSB) under aerobic conditions at 37°c overnight. Aliquots of these cultures were used for different phenotypic determinations.

#### Plate competition assays

Overnight cultures of SA isolates were adjusted to an optical density at 600 nm (OD_600nm_) of 1 and inoculated as a lawn culture into tryptic soy agar (TSA) plates. A 5-μL drop of a *P. aeruginosa* PAO1 culture, adjusted to an OD_600nm_of 10, was placed at the center of each plate. After drying, the plates were incubated overnight at 37°C. The diameter of growth inhibition zone and colony diameter were measured in each plate using ImageJ software.^44^

#### Virulence factors

For staphyloxanthin pigment quantification, 1 mL pellet of SA TSB cultures was resuspended in 1 mL of methanol and incubated with agitation overnight at 37°C in the dark. Tubes were centrifuged for 1 min at 13500 rpm and the absorbance at 462 nm of the supernatant was measured in a spectrophotometer. For other determinations, 5 μL drops of SA isolates cultures were placed on the corresponding plates and incubated overnight at 37°C. DNAse agar (Britania) was used to determine extracellular DNAse activity following manufacturer instructions. Hemolytic activity was analyzed in sheep blood agar 5%(v/v). Three biological replicates were measured for each isolate. For all tests, the plates were prepared always with 20 mL of medium and the degradation halo or clear zone was measured in each case three times per halo, and the colony diameter was measured for normalization using the ImageJ software.^44^

Quantitative hemolysis assay was performed following Olchowik-Grabarek et al.^46^ Briefly, sterile sheep blood was centrifuged at 2600 × g at 4 °C and the buffy coat was removed. Erythrocytes were washed twice with 0.9% NaCl and resuspended to a final concentration of 2% in 0.9% NaCl. Randomly selected isolates were cultivated overnight as described above. Cultures were centrifuged, and 100 μL of supernatant was added to 2 mL of the 2% erythrocyte suspension and incubated for 60 min at 37 °C. Subsequently, 0.5 mL of each sample was mixed with 1 mL of 0.9% NaCl, centrifuged, and hemoglobin release was measured at 540 nm. The positive control consisted of erythrocytes treated with 1 mL of distilled water. Controls included erythrocytes without additives and erythrocytes incubated with 100 μL of non-inoculated TSB. USA300 strain was used as an additional control.

#### Biofilms assay

Biofilm formation was analyzed in 96-multiwell polystyrene plates using crystal violet assay. Briefly, SA liquid cultures were inoculated at an initial OD_600nm_ of 0.025 in TSB supplemented with 0.5% KNO_3_ to support microaerobic reduction of nitrate that is relevant in the infection context.^47^ Plates were incubated overnight at 37°C. The supernatant’s absorbance was measured at 595 nm to determine the planktonic growth, and the adherent biomass was stained with crystal violet solution for 20 min. After incubation, the plate was washed, and the remaining dye was solubilized using absolute ethanol. The absorbance at 570 nm was determined using BMG FLUOstar Optima Microplate Reader Fluorescence Absorbance Spectrometer.^48^ Biofilm index was calculated as the ratio between A_570nm_/A_595nm_ to normalize adherent biomass to planktonic growth.^49^ *S. aureus* USA300 and non-inoculated medium were used as controls.

#### Molecular typing

Pulsed-field gel electrophoresis (PFGE) analysis of SmaI-digested chromosomal DNA was performed on selected *S. aureus* isolates and interpreted as previously described.^29^ *spa* typing was conducted on representative isolates from each PFGE-defined subtype as previously reported, and *spa* types were assigned using the RIDOM SpaServer (http://spaserver.ridom.de/). The resulting *spa* types were used to infer putative multilocus sequence types (MLST) based on previously established associations between spa types and MLST sequence types (STs).^29^^,^^33^^,^^50^ When STs could not be reliably determined from the spa database or from the published literature, including spa types without an associated ST in the RIDOM SpaServer (t1987, t665, t1184, t6587, and t4255) or spa types associated with more than one ST within the same CC (t002 and t065), as well as isolates with unassigned spa types, the genetic background of the isolates was determined by MLST as previously described by Barcudi et al.^33^ Allele numbers, sequence types (ST) and clonal complexes (CC)s were assigned using the PubMLST *Staphylococcus aureus* database https://pubmlst.org/organisms/staphylococcusaureus. The specific sequence of the *sau1*-*hsdS1* gene characteristic of CC398, was also detected by PCR following the protocol of Stegger et al.^41^

#### Antibiotics susceptibility assays

Antibiotic susceptibility was analyzed on Mueller-Hinton agar (M-H, Oxoid) plates following standardized procedure.^51^ Briefly, SA TSB cultures were adjusted to McFarland turbidity of 0.5 and 100 μL of this suspension was inoculated as a lawn culture into M-H plates. A disk of cefoxitin (FOX; 5 μg) was placed at the center of each plate, and the inoculated plates were incubated overnight at 37°C. The diameter of the growth inhibition zone was measured using ImageJ software.^44^ Antibiotic susceptibility category was interpreted according to the Clinical and Laboratory Standards Institute (CLSI) breakpoints.^52^

#### Survival assays in presence of PA and ciprofloxacin

Minimum inhibitory concentration (MIC) assays for ciprofloxacin were performed following standard protocols. Briefly, serial 2-fold dilutions of ciprofloxacin (Sigma) were prepared in sterile microplates using cation-adjusted Mueller–Hinton broth (Thermo-Fisher). Each well was inoculated with 1 × 10^5^ CFU of the corresponding SA isolate and bacterial growth was monitored by measuring OD_600nm_ for 12 h at 37 °C. One MRSA isolate and one MSSA isolate, both exhibiting a ciprofloxacin MIC of 0.5 μg/mL, were selected for subsequent experiments (Table S6; Figure S9).

For coculture experiments, *P. aeruginosa* PA14 and the selected SA isolates (FO and EB) were pre-cultured on TSA prior to inoculation. Mono- and co-cultures were prepared as indicated in Table S1. Cultures were incubated on Muller-Hinton broth in microaerobic conditions at 37°C for 16 h, and CFU/mL were determined by plating on Oxacillin Resistance Screening Agar Base (Oxoid) plates without oxacillin. The relative proportions of MRSA and MSSA colonies within cocultures were further assessed by replica plating onto agar plates containing 4 μg/mL oxacillin (Sigma) (Figure S9).

### Quantification and statistical analysis

For phenotypical analysis between different isolates groups (SA_M_, SA_T_ and SA_C_) was used One-way ANOVA with Tukey’s test. For figures using asterisks ∗*p* value > 0.05; ∗∗*p* value > 0.005 and ∗∗∗*p* value > 0.0005.

Continuous variables were expressed as median and interquartile range (IQR). Association between phenotypes and chronic PA infection status was performed using Mann-Whitney’s test. Correlations between phenotypes were evaluated with Spearman’s rank test. In addition, principal component analysis of the phenotypes was carried out using the *FactoMineR* package.^43^ The principal components obtained were then used to perform hierarchical clustering analysis with Ward’s method.^53^ Association between the clustering and clinical data, such as patient, chronic PA infection, modulator treatment or co-isolation with *Candida* spp. or *Aspergillus* spp., was then analyzed using Fisher’s exact test. Proportions of MRSA of different sampling dates were compared using Fisher’s exact test. All statistical analyses were performed with GRAPH-PAD PRISM version 8.0.2 (Dotmatics, Boston, MA, USA) for Windows and R version 4.4.2.^53^ and a *p* < 0.05 was considered as statistically significant.

## Supplemental information

Document S1. Figures S1–S10 and Tables S2–S6

Table S1. Patient characteristics Different characteristics from all patients included in this study
