# Supplementary material for: Microenvironment shapes Staphylococcus aureus phenotypes in children with cystic fibrosis over time
Source: iScience. 2026 Oct 1;29(10):117708. doi: 10.1016/j.isci.2026.117708 (PMC13657234; doi:10.1016/j.isci.2026.117708)
Supplement: Supplementary file 1 [file mmc1.pdf]

iScience, Volume *29*

Supplemental information

Microenvironment shapes *Staphylococcus aureus* phenotypes in children with cystic fibrosis over time

Stefania A. Robaldi, Rosana Pereda, Maria Constanza Pautasso, Enrique Blasko, Ailen Fretes, Trinidad Jaureguialzo, Mateo Diaz Appella, Mariana B. Galeano, Maria Jose Rial, Silvina Smith, Eugenia Ginestet, Norma Gonzalez, Javier M. González, Andrea Smania, Alejandro J. Vila, Claudia Sola, Pablo M. Cassanelli, and Paula M. Tribelli

Supplementary Fig.1 Principal component analysis based on the mean values of the different phenotypes. A. Dimensions 1 and 2. B. Dimensions 1 and 3

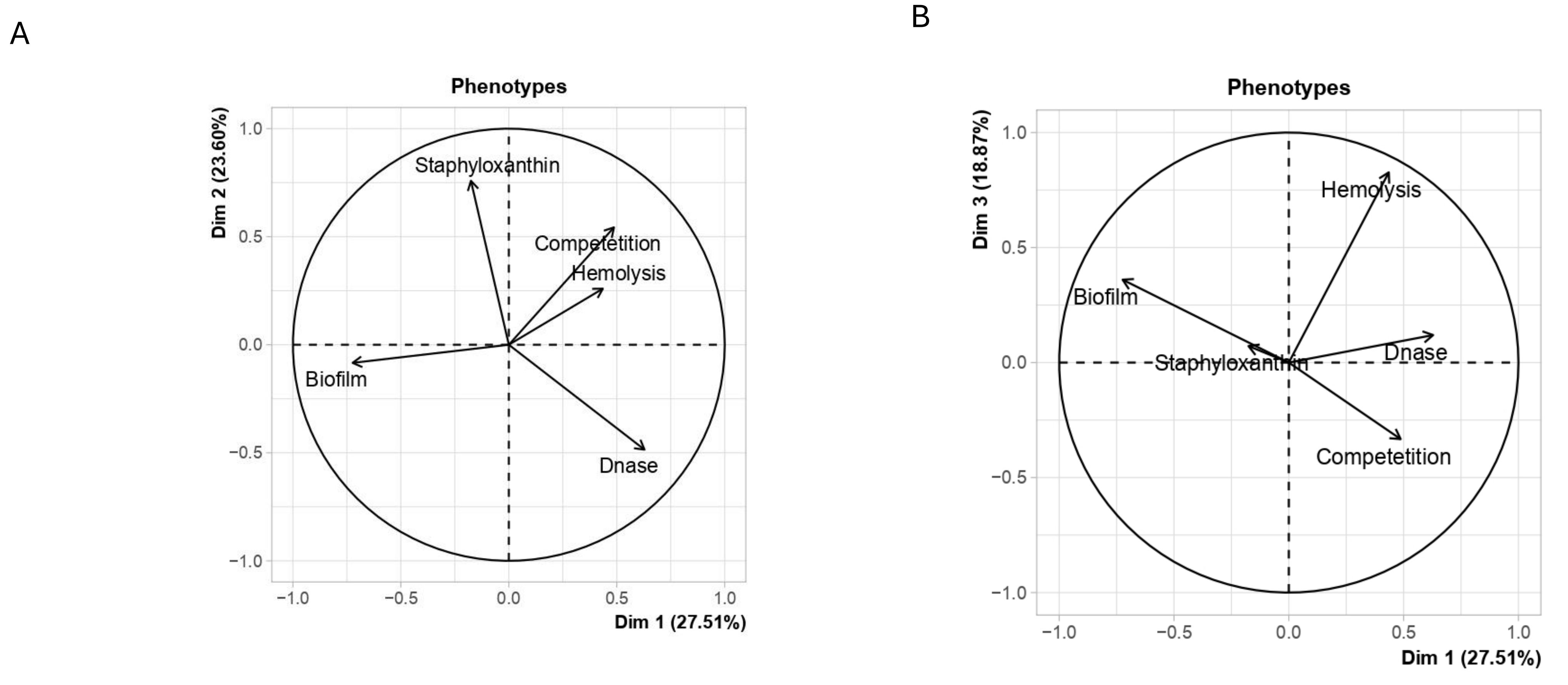

Supplementary Figure 2 **S2**: Temporal dynamics of the phenotype values in SA isolates from P4

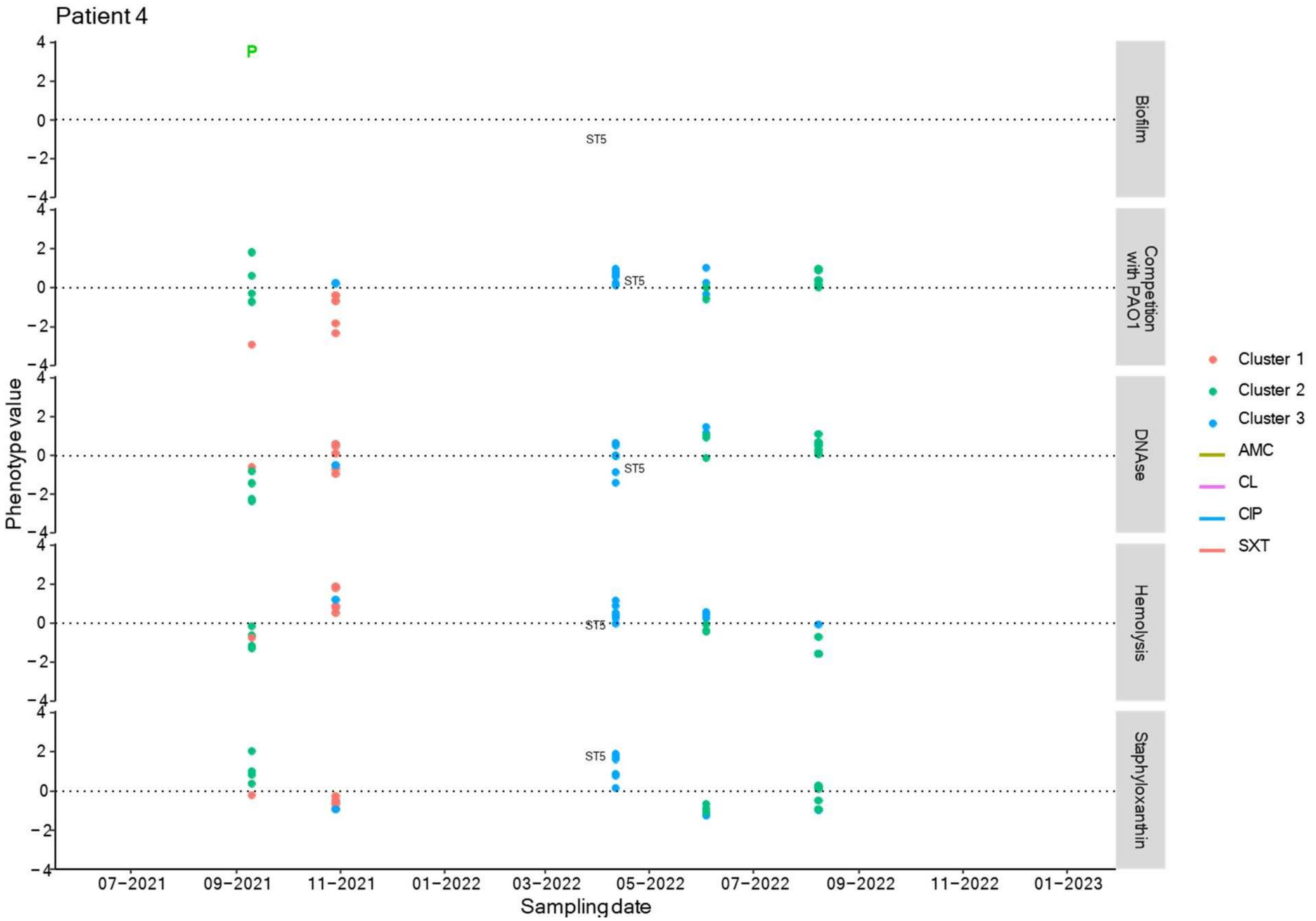

Supplementary Figure 3

S3: Temporal dynamics of the phenotype values in SA isolates from P5

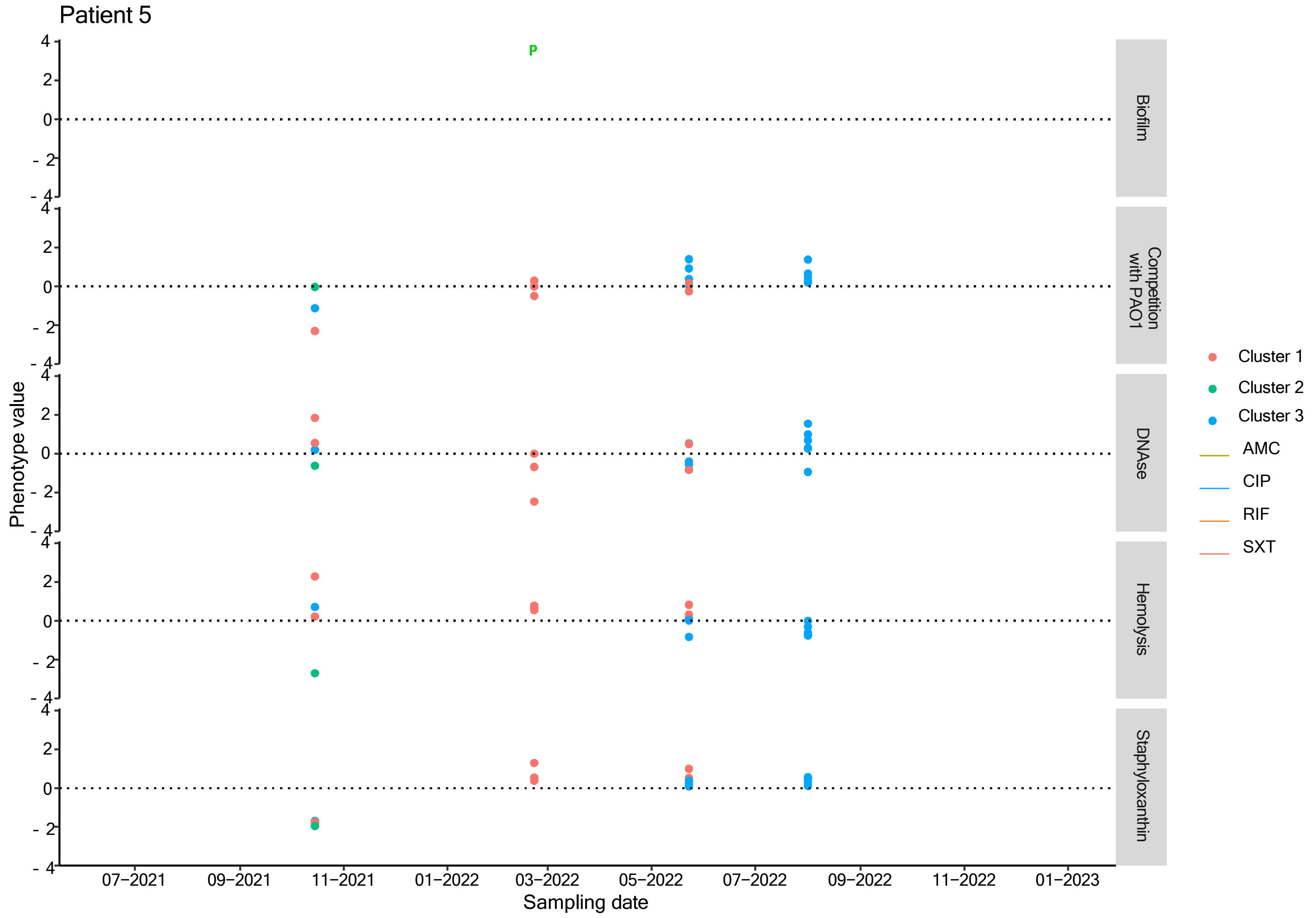

Supplementary Figure 4

S4: Temporal dynamics of the phenotype values in SA isolates from P6

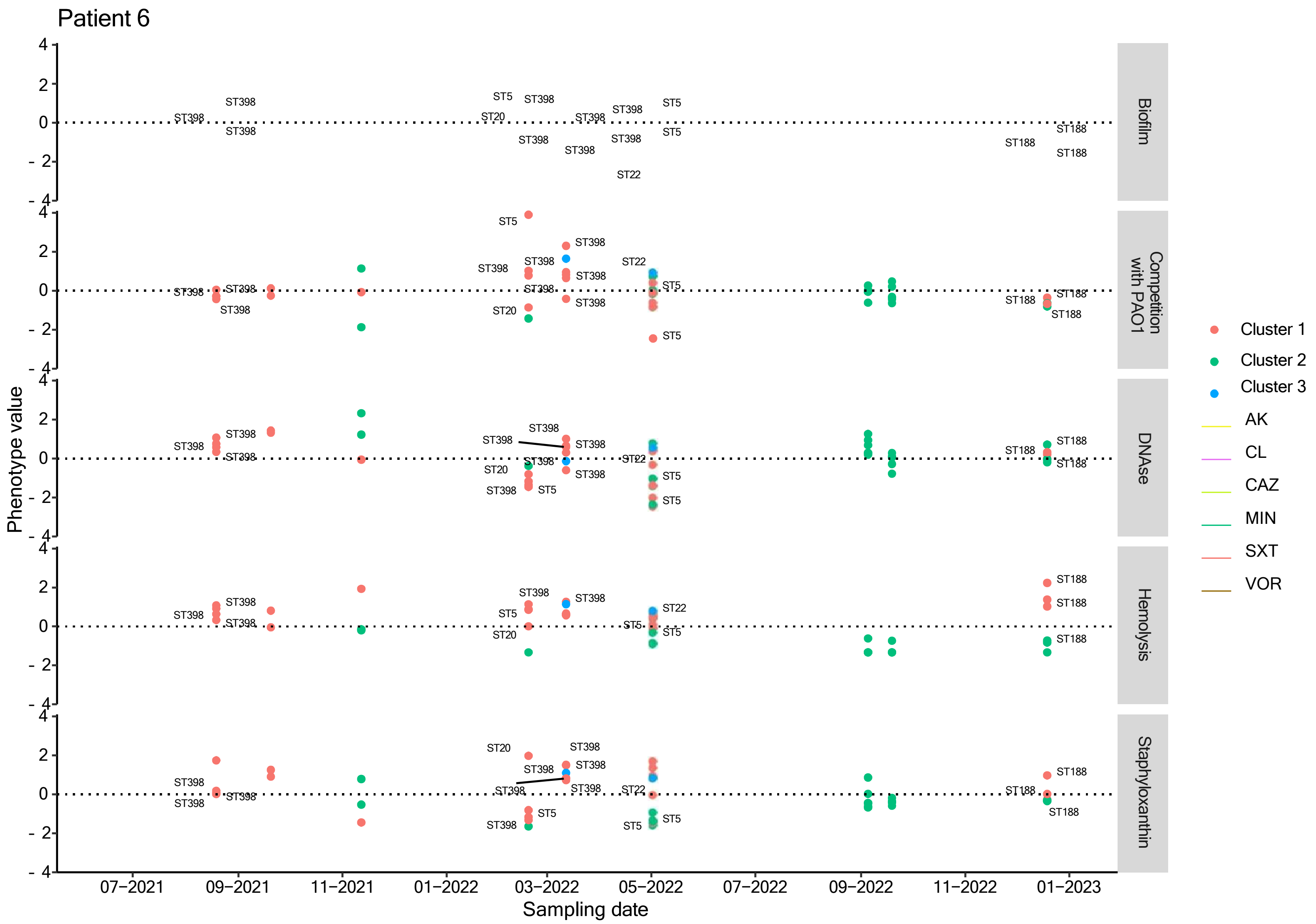

Supplementary Figure 5 **S5**: Temporal dynamics of the phenotype values in SA isolates from P13

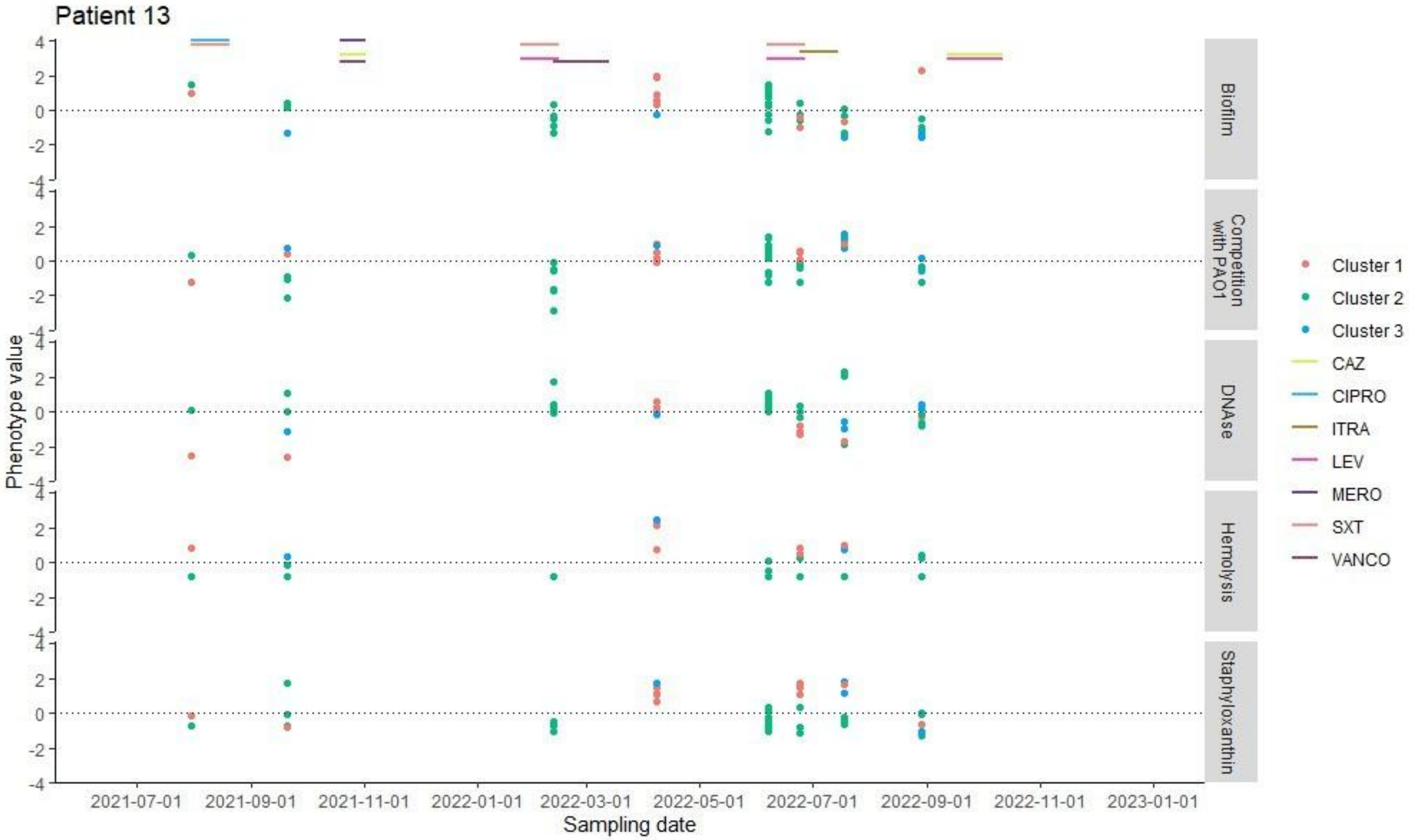

Supplementary Figure 6

S6: Temporal dynamics of the phenotype values in SA isolates from P16

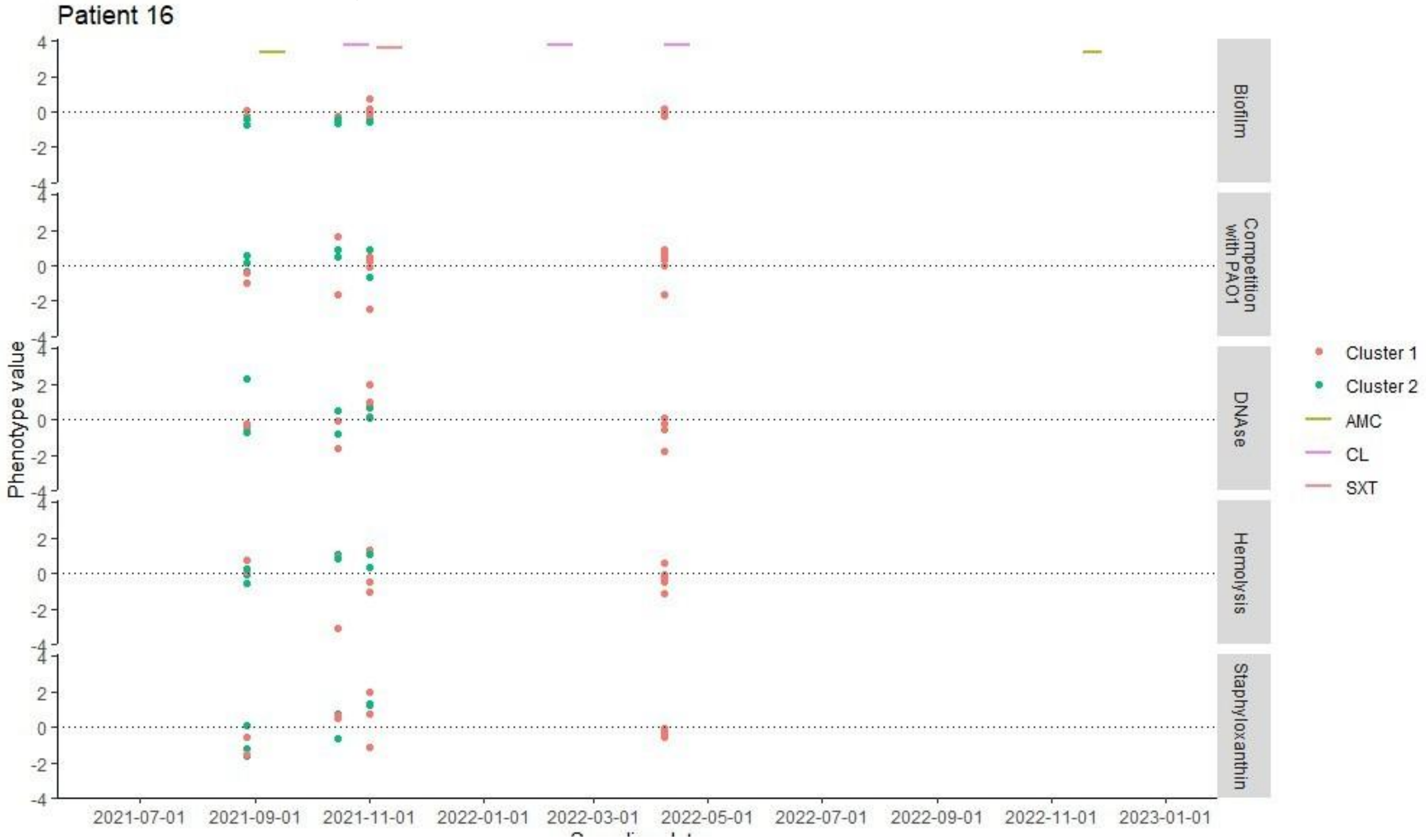

Supplementary Figure 7 **S2**: Temporal dynamics of the phenotype values in SA isolates from P17

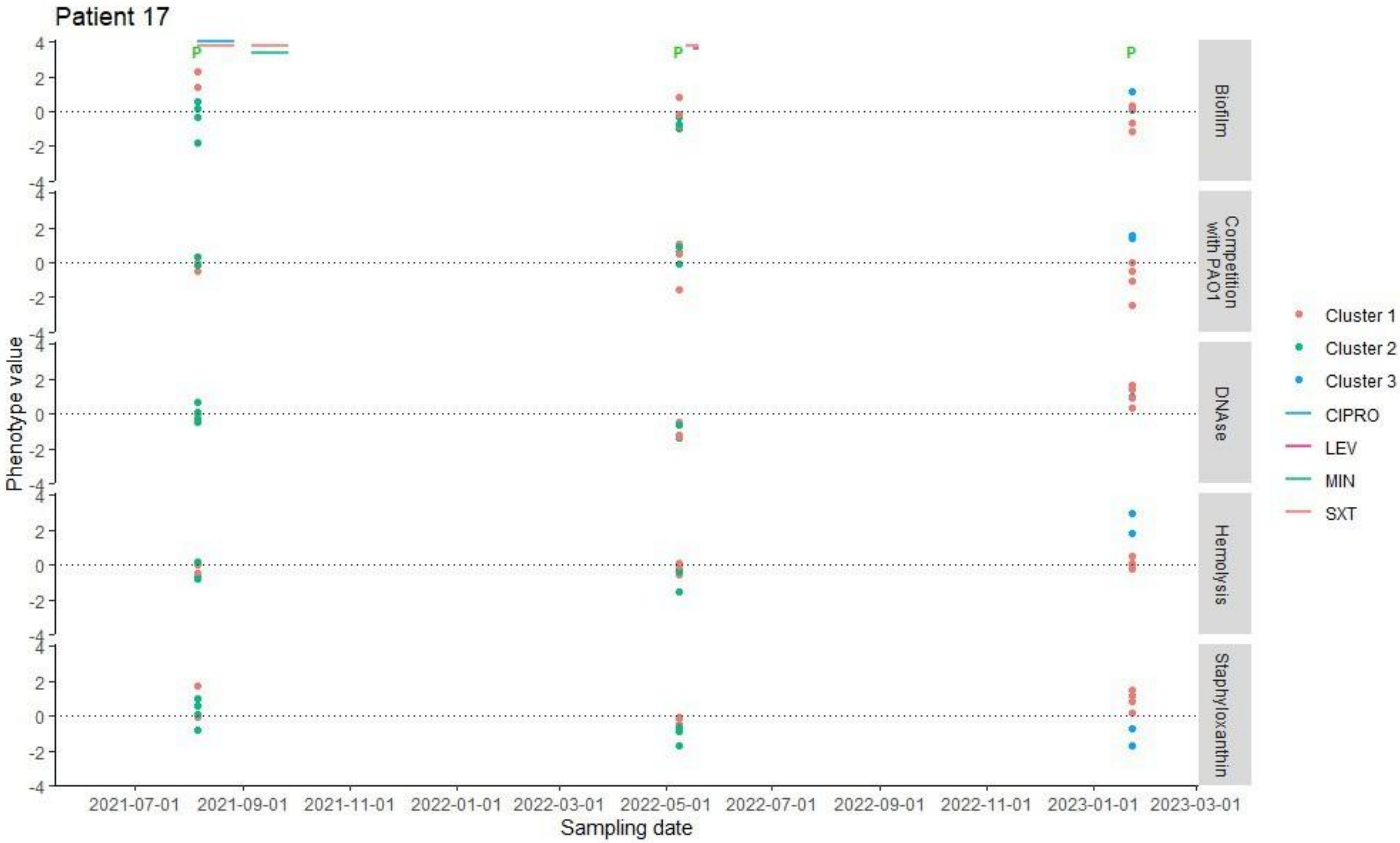

Supplementary Figure 8 **S8**: Temporal dynamics of the phenotype values in SA isolates from P18

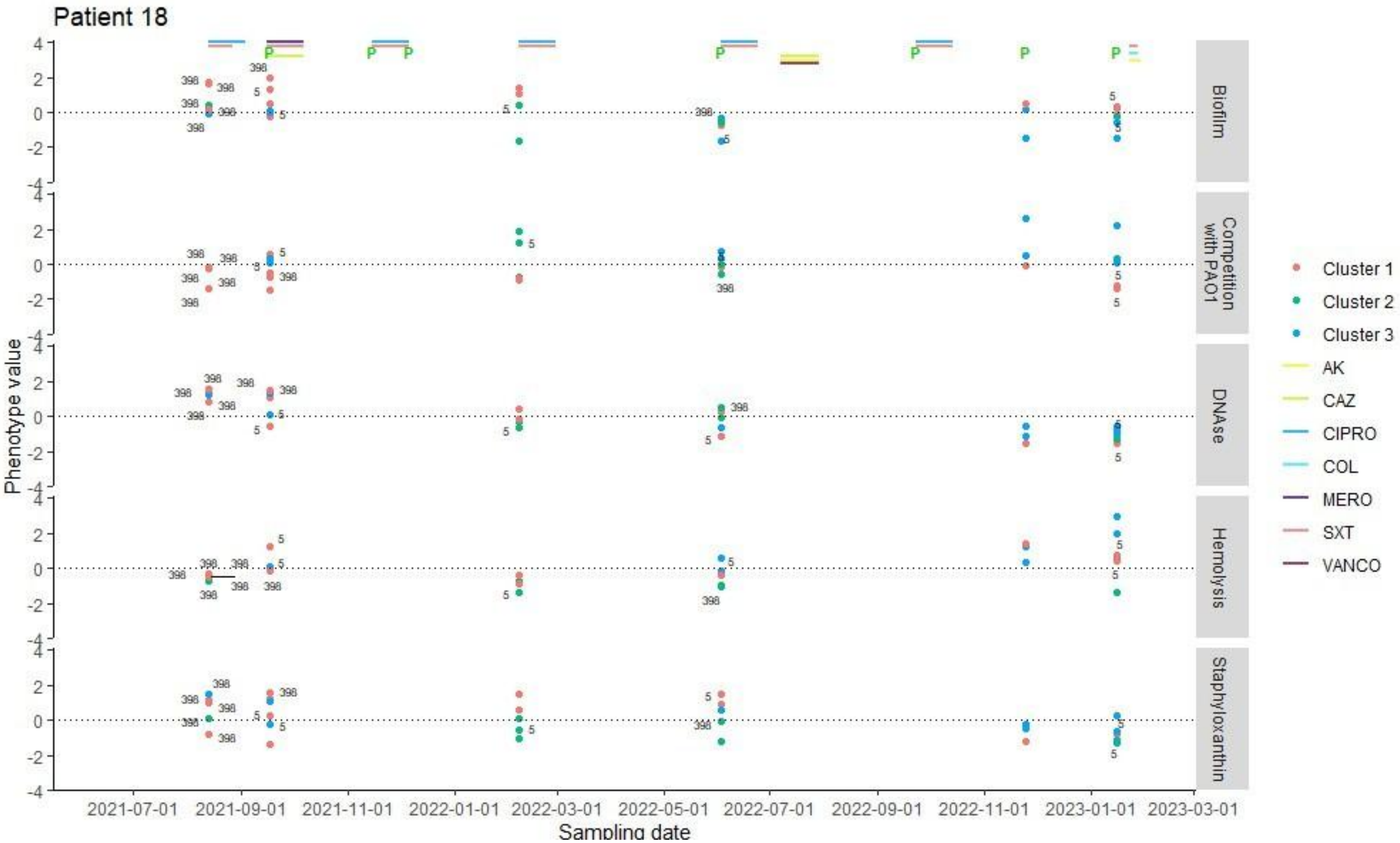

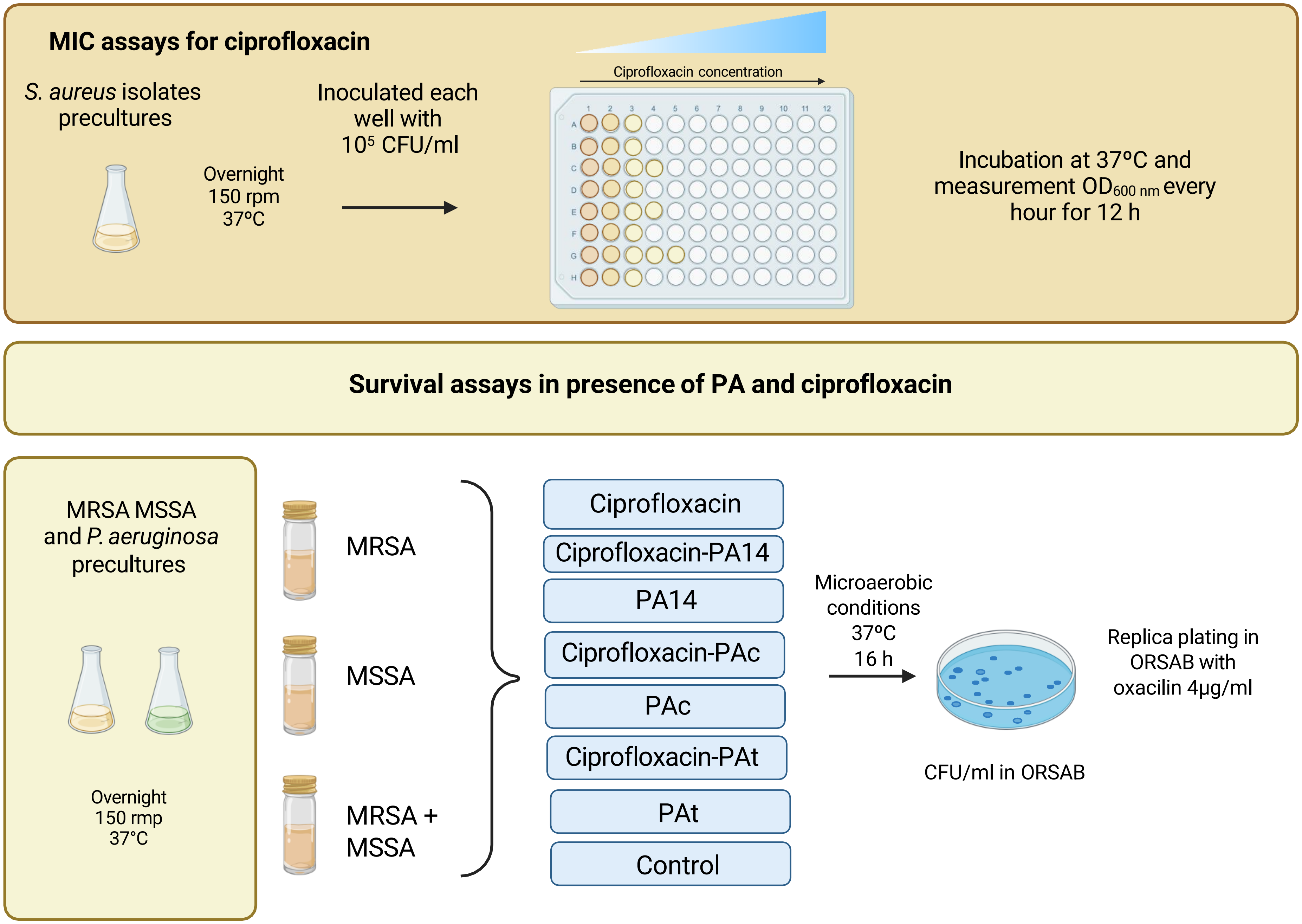

Supplementary Figure S9: Survival assays in presence of PA and ciprofloxacin. MIC assays for ciprofloxacin were performed on selected isolates. Two isolates, one MRSA and one MSSA, were evaluated in mono- and co-cultures with PA14 under ciprofloxacin exposure. The proportion of MRSA was determined by replica plating in ORSAB with oxacillin 4 µg/ml

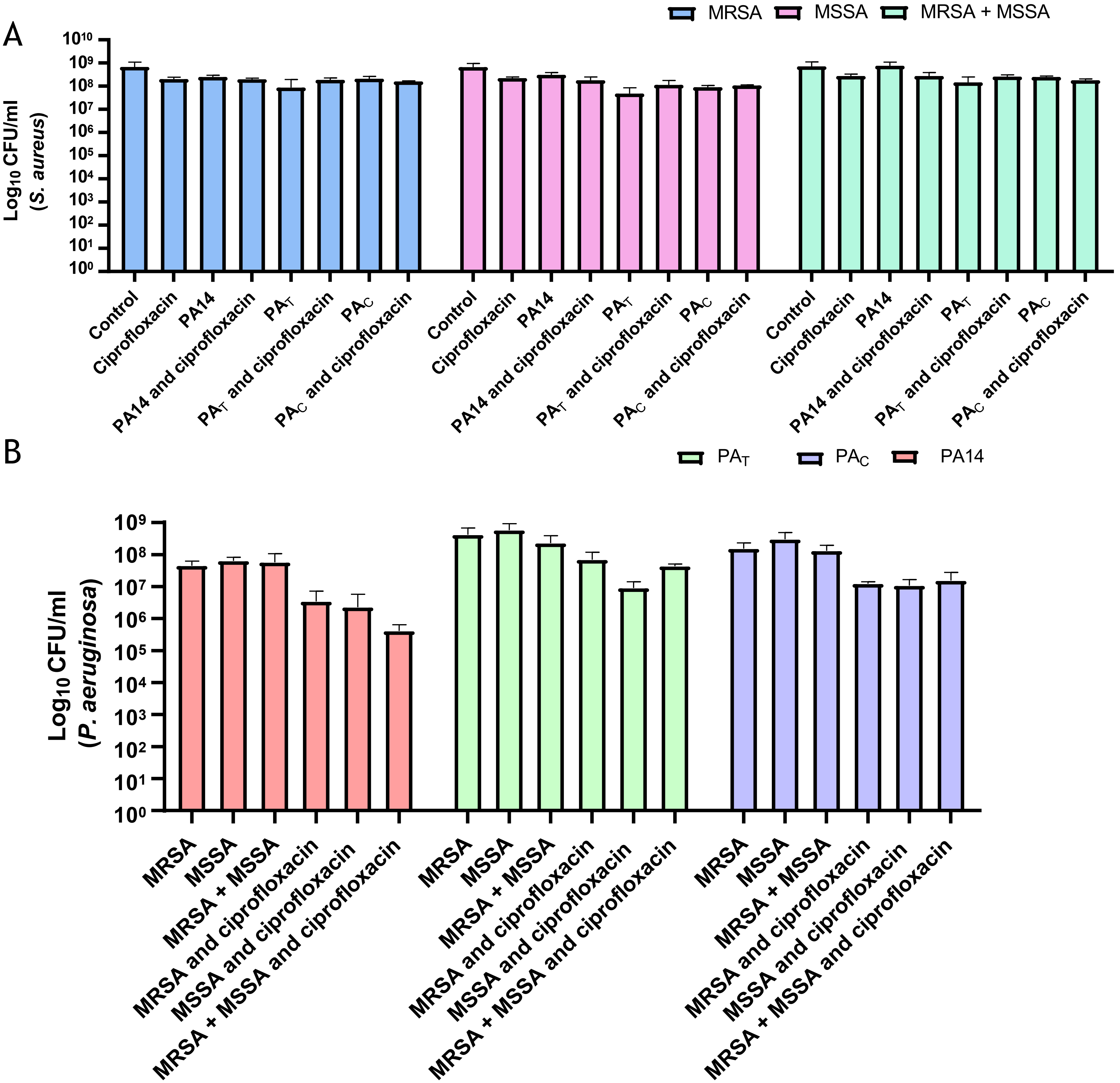

Suppplementary figure S10 Bacterial survival under ciprofloxacin tratament

A) *Staphylococcus aureus* survival (Log10CFU/ml) B) *Pseudomonas aeruginosa* survival (Log10CFU/ml). PA_T and PA_C correspond to different *P. aeruginosa* isolates, obtained from one transiently infected monoinfected patient and one coinfected patient, respectively. Both species were exposed to the same concentration of ciprofloxacin.

Table S2: *S. aureus* isolates a reference comparison of hemolysis assessed on blood agar plates (hemolytic halo–to–colony ratio, measured in cm) and in a liquid erythrocyte assay (absorbance at 540 nm; A540). Values are reported as mean and SD of three independent replicates for each isolate in both assays.

Isolate	Group	Hemolytic halo – to colony ratio (mean ± sd)	A540 (mean ± sd)
IW	SA_M	1	0.054 ± 0.008
CT	SA_M	2.20 ± 0.41	0.331 ± 0.026
AU	SA_M	1.65 ± 0.38	0.249 ± 0.038
DK	SA_M	1	0.066 ± 0.026
PP	SA_M	1.74 ± 0.02	0.240 ± 0.014
LS	SA_M	1	0.077 ± 0.008
BF	SA_T	1	0.053 ± 0.005
HT	SA_T	1.62 ± 0.50	0.256 ± 0.014
HS	SA_T	1.14 ± 0.25	0.071 ± 0.017
FR	SA_T	1	0.051 ± 0.014
PI	SA_T	1.85 ± 0.30	0.224 ± 0.007
FQ	SA_T	1	0.046 ± 0.010
HU	SA_T	1.67 ± 0.52	0.251 ± 0.015
SL	SA_C	1	0.042 ± 0.004
UM	SA_C	2.57 ± 0.31	0.267 ± 0.030
PD	SA_C	2.06 ± 0.66	0.310 ± 0.010
SK	SA_C	1	0.042 ± 0.004
HA	SA_C	1.34 ± 0.07	0.272 ± 0.010
USA300	Reference	1.54 ± 0.14	0.162 ± 0.030

Table S3. Correlation between phenotypes using Spearman's rank

Phenotype	Staphyloxantin	Hemolysis	Competence	Biofilm	DNAse
**Staphyloxantin**	-				-0.15, p<0.001
**Hemolysis**		-			0.13, p<0.001
**Competence**			-	0.13, p < 0.001	0.21, p<0.001
**Biofilm**			0.13, p < 0.001	-	-0.23, p < 0.001
**DNAse**	-0.15, p<0.001	0.13, p<0.001	0.21, p<0.001	-0.23, p < 0.001	-

Table S4. PCA score table based on the mean values of the different phenotypes

Eigenvalues and variance explained

Component	Eigenvalue	% of variance	Cumulative % of variance
Comp 1	1.3757011	27.514	27.514
Comp 2	1.1802252	23.605	51.119
Comp 3	0.9435452	18.871	69.989
Comp 4	0.8219584	16.439	86.429
Comp 5	0.6785701	13.571	100.000

Coordinates

Variable	Dim.1	Dim.2	Dim.3	Dim.4	Dim.5
Mean staphyloxanthin	-0.1753919	0.7585286	0.0717161	-0.5856725	0.2138143
log(mean biofilm)	-0.7234283	-0.0839758	0.3601417	0.3103622	0.4935309
log(mean competence)	0.4869344	0.5430106	-0.3326841	0.5071358	0.3164949
log(mean hemolysis)	0.435404	0.2593978	0.8268902	0.1649415	-0.1793962
log(mean DNase)	0.6284175	-0.4854482	0.1194733	-0.313415	0.5068814

Correlation

Variable	Dim.1	Dim.2	Dim.3	Dim.4	Dim.5
Mean staphyloxanthin	-0.1753919	0.7585286	0.0717161	-0.5856725	0.2138143
log(mean biofilm)	-0.7234283	-0.0839758	0.3601417	0.3103622	0.4935309
log(mean competence)	0.4869344	0.5430106	-0.3326841	0.5071358	0.3164949
log(mean hemolysis)	0.435404	0.2593978	0.8268902	0.1649415	-0.1793962
log(mean DNase)	0.6284175	-0.4854482	0.1194733	-0.313415	0.5068814

Squared cosine (cos^2)

Variable	Dim.1	Dim.2	Dim.3	Dim.4	Dim.5
Mean staphyloxanthin	0.0307623	0.5753656	0.0051432	0.3430123	0.0457166

log(mean biofilm)	0.5233486	0.0070519	0.1297020	0.0963247	0.2435728
log(mean competence)	0.2371051	0.2948605	0.1106787	0.2571867	0.1001690
log(mean hemolysis)	0.1895767	0.0672872	0.6837474	0.0272057	0.0321830
log(mean DNase)	0.3949085	0.2356599	0.0142739	0.0982289	0.2569288

Contribution (%)

Variable	Dim.1	Dim.2	Dim.3	Dim.4	Dim.5
Mean staphyloxanthin	2.2361	48.7505	0.5451	41.7311	6.7372
log(mean biofilm)	38.0423	0.5975	13.7462	11.7189	35.8950
log(mean competence)	17.2352	24.9834	11.7301	31.2895	14.7618
log(mean hemolysis)	13.7804	5.7012	72.4658	3.3099	4.7428
log(mean DNase)	28.7060	19.9674	1.5128	11.9506	37.8633

Table S5: Molecular characterization of selected SA isolates

Isolates with PFGE	Patient	PFGE type	RIDOM *spa* type	CC398/*sau1-hsdS1*	Sequence Type (ST)	Cluster	Sampling Date
AW-7646	P1	A1	t1987	neg	ST20	1	Jul-21
AV-7647	P1	A2	t1987	neg	ST20	1	Jul-21
AU-7648	P1	A1		neg	ST20	1	Jul-21
AT-7649	P1	B1	NA/09-02-16-34-13-17-34-23-34-34	neg	ST45	1	Jul-21
HM-7650	P1	A1		neg	ST20	1	Mar-22
HN-7651	P1	A1	t1987	neg	ST20	1	Mar-22
HÑ-7652	P1	A1		neg	ST20	1	Mar-22
HO-7653	P1	A1		neg	ST20	1	Mar-22
HP-7654	P1	A1	t1987	neg	ST20	1	Mar-22
HR-7655	P1	A1		neg	ST20	1	May-22
HU-7656	P1	A1		neg	ST20	1	May-22
HQ-7657	P1	A1	t1987	neg	ST20	1	May-22
PH-7658	P1	A1		neg	ST20	3	Aug-22
PI-7659	P1	A1	t1987	neg	ST20	1	Aug-22
PJ-7660	P1	A1		neg	ST20	1	Aug-22
SU-7661	P1	A3	t1987	neg	ST20	1	Dec-22
SW-7662	P1	A3		neg	ST20	1	Dec-22
UB-7663	P1	C1	t665	neg	ST30	1	Dec-22
UA-7664	P1	C1		neg	ST30	1	Dec-22
TY-7665	P1	C1		neg	ST30	1	Dec-22
Y-7666	P12	NT	t1451	pos	ST398	1	Aug-21
Z-7667	P12	NT		pos	ST398	3	Aug-21
FE-7669	P12	NT		pos	ST398	3	Feb-22
FF-7670	P12	NT		pos	ST398	3	Feb-22
FH-7671	P12	NT		pos	ST398	3	Feb-22
FG-7672	P12	NT		pos	ST398	1	Feb-22
PL-7673	P12	NT		pos	ST398	3	Aug-22
PM-7674	P12	NT		pos	ST398	3	Aug-22
PN-7675	P12	NT	t1451	pos	ST398	3	Aug-22
PP-7677	P12	NT		pos	ST398	3	Aug-22
AO-7678	P15	D1	t002	neg	ST5	1	Aug-21
AP-7679	P15	D1		neg	ST5	1	Aug-21
CA-7680	P15	D1		neg	ST5	1	Nov-21
CE-7681	P15	D1	t002	neg	ST5	1	Nov-21
CC-7682	P15	D1		neg	ST5	1	Nov-21
JS-7683	P15	D1		neg	ST5	2	Apr-22
JT-7684	P15	D1		neg	ST5	2	Apr-22
JV-7685	P15	D1		neg	ST5	3	Apr-22
LL-7686	P15	D1		neg	ST5	2	Jun-22
LO-7687	P15	D1		neg	ST5	2	Jun-22

LK-7688	P15	D1		neg	ST5	2	Jun-22
RA-7689	P15	D1		neg	ST5	2	Sep-22
RE-7690	P15	D1		neg	ST5	2	Sep-22
RB-7691	P15	D1		neg	ST5	2	Sep-22
RC-7692	P15	D1		neg	ST5	2	Sep-22
RD-7693	P15	D1		neg	ST5	2	Sep-22
EJ-7694	P18	NT	t1451	pos	ST398	2	Aug-21
EK-7695	P18	NT		pos	ST398	1	Aug-21
EL-7696	P18	NT		pos	ST398	3	Aug-21
EM-7697	P18	NT		pos	ST398	1	Aug-21
EN-7698	P18	NT		pos	ST398	1	Aug-21
ED-7699	P18	D2	t002	neg	ST5	1	Sep-21
EF-7700	P18	D2		neg	ST5	3	Sep-21
EI-7701	P18	NT	t1451	pos	ST398	1	Sep-21
FM-7703	P18	D2		neg	ST5	2	Feb-22
MS-7705	P18	D2		neg	ST5	1	Jun-22
MV-7706	P18	NT		POS	ST398	2	Jun-22
UL-7708	P18	D2		neg	ST5	1	Jan-23
UN-7709	P18	D2		neg	ST5	1	Jan-23
GT-7710	P9	B3	t065	neg	ST45	1	Mar-22
GU-7711	P9	B2		neg	ST45	1	Mar-22
GW-7712	P9	B3		neg	ST45	1	Mar-22
GY-7713	P9	B2		neg	ST45	1	Mar-22
GZ-7714	P9	B2	t065	neg	ST45	1	Mar-22
MN-7715	P9	NT		pos	ST398	3	May-22
MÑ-7716	P9	NT		pos	ST398	3	May-22
MO-7717	P9	NT		pos	ST398	2	May-22
MP-7718	P9	NT	t1451	pos	ST398	3	May-22
MQ-7719	P9	NT	t1451	pos	ST398	3	May-22
MR-7720	P9	NT		pos	ST398	3	May-22
OZ-7721	P9	NT		pos	ST398	3	Aug-22
PA-7722	P9	NT	t1451	pos	ST398	3	Aug-22
PB-7723	P9	NT		pos	ST398	3	Aug-22
PC-7724	P9	NT		pos	ST398	1	Aug-22
PD-7725	P9	NT	t1451	pos	ST398	3	Aug-22
PE-7726	P9	NT		pos	ST398	3	Aug-22
DW-7727	P2	E1	NA: 11-10-21-17-34-24-34-34-24-34-34-22-25	neg	ST8	1	Sep-21
DY-7728	P2	NT	t1184	pos	ST398	1	Sep-21
EC-7729	P2	E1		neg	ST8	3	Sep-21
FQ-7730	P2	C2	t665	neg	ST30	2	Feb-22
FO-7731	P2	C2		neg	ST30	1	Feb-22
FT-7732	P2	C2		neg	ST30	1	Feb-22
NZ-7733	P2	C2		neg	ST30	3	Sep-22

OB-7734	P2	C2	t665	neg	ST30	3	Sep-22
OA-7735	P2	C2		neg	ST30	3	Sep-22
RQ-7736	P2	C2		neg	ST30	3	Sep-22
RT-7737	P2	C2		neg	ST30	3	Sep-22
RR-7738	P2	C2		neg	ST30	1	Sep-22
RS-7739	P2	C2	t665	neg	ST30	1	Sep-22
AH-7740	P6	NT		pos	ST398	1	Aug-21
AI-7741	P6	NT	t6587	pos	ST398	1	Aug-21
AF-7742	P6	NT		pos	ST398	1	Aug-21
GC-7745	P6	D3	t002	neg	ST5	1	Feb-22
GG-7747	P6	NT	t6587	pos	ST398	1	Feb-22
GI-7748	P6	A1	t1987	neg	ST20	1	Feb-22
HL-7749	P6	NT	t6587	pos	ST398	1	Mar-22
HI-7750	P6	NT		pos	ST398	1	Mar-22
HG-7751	P6	NT	t6587	pos	ST398	1	Mar-22
HH-7752	P6	NT		pos	ST398	1	Mar-22
HK-7753	P6	NT		pos	ST398	1	Mar-22
IV-7754	P6	D4	t4255	neg	ST5	1	May-22
IT-7755	P6	D4		neg	ST5	1	May-22
JC-7756	P6	EMRSA-15	t223	neg	ST22	3	May-22
TS-7757	P6	G1	t189	neg	ST188	1	Dec-22
TV-7758	P6	G1	t189	neg	ST188	2	Dec-22
TX-7759	P6	G1	t189	neg	ST188	1	Dec-22

NT: PFGE non-typeable strain

Table S6. Survival assays in presence of PA and ciprofloxacin.

Isolate (*S. aureus*)	Co-culture with PA14	Ciprofloxacin 0.250 µg/ml	Inoculum of S. aureus (CFU/ml)	Inoculum of *P. aeruginosa* (CFU/ml)	Ratio (SA:PA)
MSSA	Yes	No	$1x10^8$	NA	NA
MRSA	Yes	No	$1x10^8$	NA	NA
MRSA + MSSA	Yes	No	$1x10^8$	NA	NA
MSSA	Yes	Yes	$1x10^8$	$1x10^8$	1:1
MRSA	Yes	Yes	$1x10^8$	$1x10^8$	1:1
MRSA + MSSA	Yes	Yes	$1x10^8$	$1x10^8$	1:1:1
MSSA	No	Yes	$1x10^8$	NA	NA
MRSA	No	Yes	$1x10^8$	NA	NA
MRSA + MSSA	No	Yes	$1x10^8$	NA	NA
MSSA	No	No	$1x10^8$	NA	NA
MRSA	No	No	$1x10^8$	NA	NA
MRSA + MSSA	No	No	$1x10^8$	NA	NA

NA: Not applied
